# Mechanistic insights and *in vivo* efficacy of thiosemicarbazones against methicillin-resistant *Staphylococcus aureus*

**DOI:** 10.1016/j.jbc.2024.107689

**Published:** 2024-08-17

**Authors:** Avery Gaudreau, David W. Watson, Ronald S. Flannagan, Paroma Roy, Chenfangfei Shen, Ahmed Abdelmoneim, William N. Beavers, Elizabeth R. Gillies, Omar M. El-Halfawy, David E. Heinrichs

**Affiliations:** 1 Department of Microbiology and Immunology, The University of Western Ontario, London, Ontario, Canada; 2 Department of Chemistry, The University of Western Ontario, London, Ontario, Canada; 3 Department of Comparative Biomedical Sciences, School of Veterinary Medicine, Louisiana State University, Baton Rouge, Louisiana, USA; 4 Louisiana Animal Disease Diagnostic Laboratory, Louisiana State University, Baton Rouge, Louisiana, USA; 5 Department of Pathobiological Sciences, Louisiana State University School of Veterinary Medicine, Baton Rouge, Louisiana, USA; 6 Department of Chemical and Biochemical Engineering, The University of Western Ontario, London, Ontario, Canada; 7 Department of Chemistry and Biochemistry, University of Regina, Regina, Saskatchewan, Canada; 8 Department of Microbiology and Immunology, Faculty of Pharmacy, Alexandria University, Alexandria, Egypt

**Keywords:** MRSA, *S. aureus*, antibiotic, MOA, infection model, thiosemicarbazones

## Abstract

*Staphylococcus aureus* poses a significant threat in both community and hospital settings due to its infective and pathogenic nature combined with its ability to resist the action of chemotherapeutic agents. Methicillin-resistant *S. aureus* (MRSA) represents a critical challenge. Metal-chelating thiosemicarbazones (TSCs) have shown promise in combating MRSA and while previous studies hinted at the antimicrobial potential of TSCs, their mechanisms of action against MRSA are still under investigation. We screened a chemical library for anti-staphylococcal compounds and identified a potent molecule named R91 that contained the NNSN structural motif found within TSCs. We identified that R91 and several structural analogs exhibited antimicrobial activity against numerous *S. aureus* isolates as well as other Gram-positive bacteria. RNAseq analysis revealed that R91 induces copper and oxidative stress responses. Checkerboard assays demonstrated synergy of R91 with copper, nickel, and zinc. Mutation of the SrrAB two-component regulatory system sensitizes *S. aureus* to R91 killing, further linking the oxidative stress response to R91 resistance. Moreover, R91 was found to induce hydrogen peroxide production, which contributed to its antimicrobial activity. Remarkably, no mutants with elevated R91 resistance were identified, despite extensive attempts. We further demonstrate that R91 can be used to effectively treat an intracellular reservoir of *S. aureus* in cell culture and can reduce bacterial burdens in a murine skin infection model. Combined, these data position R91 as a potent TSC effective against MRSA and other Gram-positive bacteria, with implications for future therapeutic development.

*Staphylococcus aureus* is a significant cause of community and hospital acquired infections, owing to the emergence of strains that display enhanced transmission, virulence, and/or resistance to multiple antibiotics (1). This pathogen is capable of causing a wide spectrum of infections, ranging from skin and soft tissue infections to life-threatening conditions such as bacteremia and endocarditis (1). Furthermore, the remarkable ability of *S. aureus* to acquire and upregulate antimicrobial resistance genes, coupled with the overuse and misuse of antibiotics, has led to a critical threat (2). Methicillin-resistant *S. aureus* (MRSA) is of particular concern. Beyond β-lactams, MRSA displays resistance to various antibiotic classes through mechanisms involving target modification, drug modification, and decreased cell membrane permeability (3). This abundance of resistance mechanisms has resulted in approximately 95% of MRSA clinical isolates being resistant to first-line antibiotics (4). Strains resistant to vancomycin and daptomycin, or “last resort” antibiotics, further complicate treatment, underscoring the urgency of discovering new antibiotics to address the growing threat of multidrug-resistant MRSA (MDR-MRSA) (2).

Metal ion homeostasis plays a critical role in bacterial infection and pathogenesis (5, 6). Metals such as iron, zinc, and copper are essential for various bacterial processes, including metabolism, DNA replication, and defense against oxidative stress induced by reactive oxygen species (ROS) (5, 6). Consequently, pathogens like *S. aureus* rely on tightly regulated metal ion acquisition and utilization systems to thrive within the host environment and evade host nutritional immunity mechanisms (7, 8).

Metal chelators, particularly thiosemicarbazones (TSCs), have emerged as a promising solution to combat MDR-MRSA (9, 10, 11, 12, 13). Characterized by their NNSN metal-binding structural motif (11), TSCs have been shown to interact with various metals, most commonly copper, and their antibacterial activity has often been demonstrated to be copper-dependent (11, 12). These chelators can mimic the host's nutritional immunity strategy by sequestering or chelating essential metals from bacteria, hindering vital bacterial processes that rely on these metals (11). Additionally, complexation with copper can deliver toxic levels of the metal to bacterial cells and increase membrane permeability to disrupt cell integrity and function (12).

Importantly, TSCs offer stability and selectivity in metal binding, reducing off-target toxic effects, and enhancing the specificity for bacterial targets (14, 15). Prior studies have reported the antimicrobial potential of TSCs (9, 10, 11, 12, 13, 14, 15, 16, 17, 18, 19), but their full spectrum of mechanisms of activity against MRSA remains an area of active research.

Here we report the identification of a TSC (named R91) that is highly effective in the low micromolar concentration range against MRSA, using high-throughput screening of bioactives from the Maybridge compound collection. Assessment of the activity of R91 and structurally related analogs against *S. aureus* isolates and other bacterial species demonstrated these compounds possess broad-spectrum antimicrobial activity against Gram-positive bacteria. RNAseq identified R91-induced copper and oxidative stress responses in *S. aureus*, highlighting its metal-associated antibacterial activities. Checkerboard assays demonstrated enhanced antibacterial activity of R91 when combined with copper, nickel, and zinc. Furthermore, we found that mutation of the SrrAB two-component regulatory system, which is involved in the response to oxidative stress, sensitized *S. aureus* to R91, providing an important link between sensing of ROS by SrrAB and resistance to R91. Importantly, despite numerous attempts using different strategies, we failed to identify *S. aureus* mutants with elevated resistance to R91. Finally, after demonstrating that R91 was nontoxic to eukaryotic cells (macrophages) at concentrations well above the minimum inhibitory concentration (MIC), we showed that R91 was effective in clearing intracellular macrophage infections. In subcutaneous (SQ) infections of mice, R91 reduced both lesion size and bacterial burden. Together, these findings advance our understanding of R91 as a representative TSC, offering important insight into its mechanisms of action and potential therapeutic applications.

## Results

### High-throughput screening and identification of an anti-staphylococcal TSC

**Figure 1:**
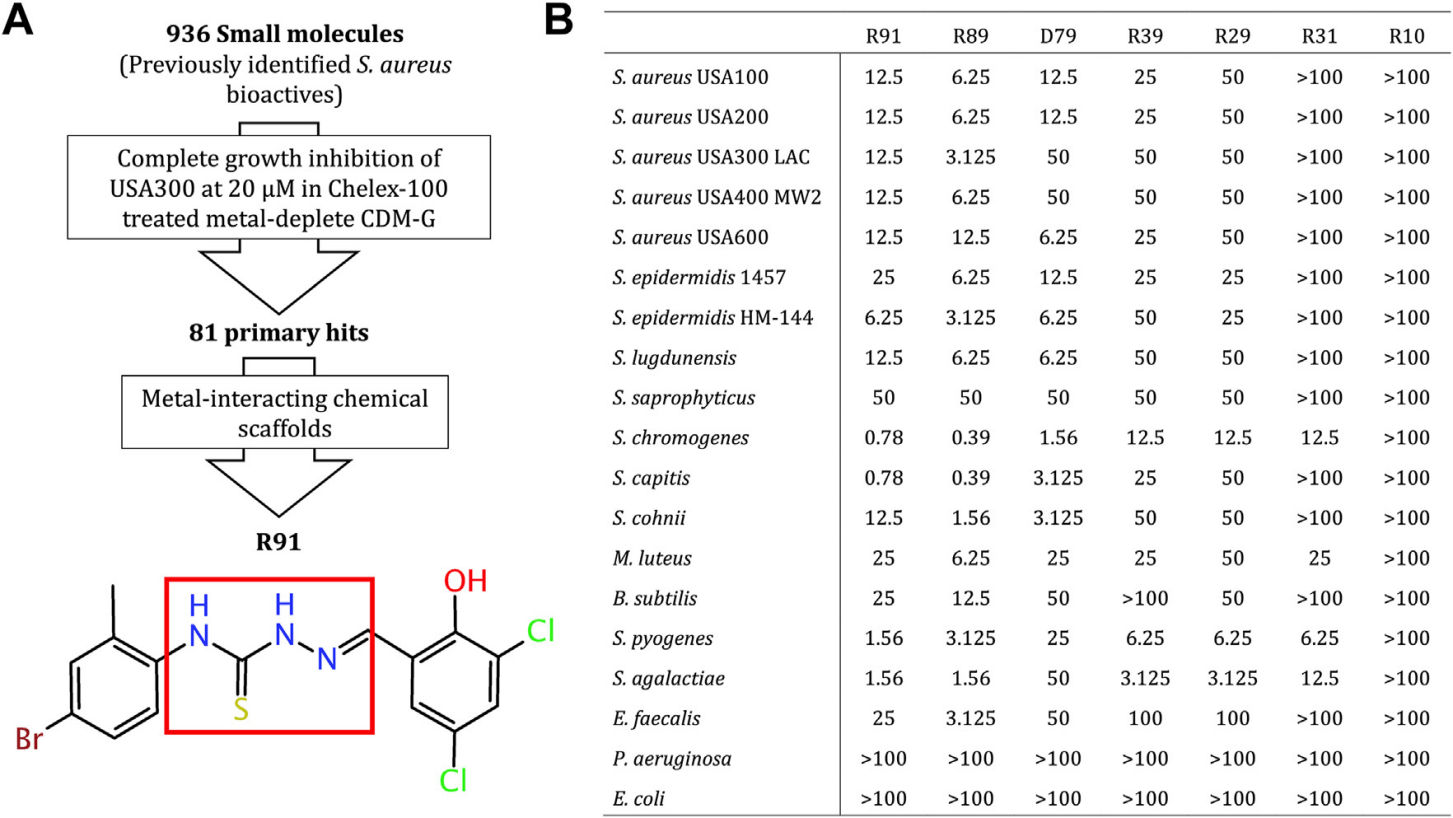
**High-throughput screening identifies R91, a potent TSC with activity against MRSA USA300 LAC.***A*, the workflow undertaken in this study that identified R91 and the chemical structure of R91. A red box has been placed around the core NNSN structure, characteristic of all TSCs. *B*, MIC (μM) of TSCs against various *Staphylococcus aureus* isolates and other bacterial species was determined using the concentration of drug that completely inhibited growth as determined by A_600_ readings after 24 h of incubation in THY for *Streptococcus*, TSB for all other Gram-positive species, and LB for Gram-negatives. Strain designations and descriptions for the various bacterial species listed can be found in Table S1.

A high-throughput screen of 50,000 diverse small molecules sourced from the Maybridge a library against a methicillin-sensitive *S. aureus* (MSSA) strain in tryptic soy broth (TSB) at 10 μM previously identified *S. aureus* bioactives showing partial to complete growth inhibition of the tested MSSA under the screen conditions (20). Herein, we screened 936 of these bioactives against the *S. aureus* USA300 LAC strain, a widely circulating community-acquired MRSA strain in North America (21), at 20 μM in duplicate (Fig. 1*A*). We sought to enrich for compounds with the ability to enhance metal-related toxicity or interfere with determinants involved in metal ion homeostasis, which are more likely expressed under metal ion limitation rather than under metal-replete conditions (6). As such, we conducted the screen in a Chelex-100 resin-treated metal-deplete chemically defined medium containing glucose (CDM-G) (22), identifying 81 compounds that completely inhibited growth of *S. aureus* USA300 LAC. We filtered the hits based on chemical scaffolds known to interact with metals. We then prioritized R91 (see Fig. 1*A*), a TSC with an MIC of 20 μM against USA300 LAC in metal-deplete CDM-G, for further follow up. Importantly, the structure of R91 is unique compared to other reported small-molecule modulators of metal homeostasis (9, 10, 11, 12, 13, 14, 15, 16, 17, 18, 19, 23, 24, 25, 26). The ability of various TSC compounds to chelate metals and their potential as antimicrobials has been previously established, yet their mechanism of action remains undefined (9, 11, 12, 13, 16). In addition to R91, we selected other TSC structural analogs D79, R29, R39, R89, R31, and R10 (see Fig. S1), for subsequent testing to infer preliminary structure activity relationships.

### Antibacterial activities of TSCs on assorted bacteria

To assess the effect of R91 on bacterial viability outside of the above screening conditions, we evaluated the bactericidal activity of this TSC against USA300 LAC. Bacteria were co-inoculated with R91 at various concentrations in PBS before being incubated for 24 h at 37 °C. Subsequently, the number of viable bacteria was determined by drop plating onto tryptic soy agar (TSA) that was then incubated for an additional 24 h. These data revealed that, under the conditions used, only at concentrations ≥100 μM was R91 capable of significantly reducing *S. aureus* viability (Fig. S2*A*). To assess the efficacy of R91 against actively dividing cells, we substituted Mueller Hinton Broth (MHB) for PBS in our method. Similar trends were observed in MHB, with R91 inhibiting growth below 100 μM and reducing viability at concentrations ≥100 μM (Fig. S2*B*). Notably, although 100 μM of R91 decreased bacterial viability in MHB, the TSC was less effective than in PBS, suggesting a particular ability of R91 in targeting nondividing cells.

To further assess the utility of R91 and the other selected TSCs as antimicrobials, we assessed the antimicrobial potential of these compounds against a variety of clinically relevant bacteria (Fig. 1*B*). Of the TSCs examined, R89, R91, and D79 exhibited substantial antimicrobial activity against most of the Gram-positive bacteria examined, with MIC values ranging from less than 1 μM to 25 μM, except for *Staphylococcus saprophyticus,* which was more resistant. Interestingly, the MIC for D79 against representative USA300 and USA400 isolates was significantly higher than that of R89 and R91. In contrast, R10 and R31 exhibited no antimicrobial activity against most Gram-positive and Gram-negative bacteria, although notably, R31 was found to be effective against *S. chromogenes, S. pyogenes, S. agalactiae*, and *M. luteus*. R89 was identified as the analog with activity most comparable to R91 and thus was selected, along with R91, for stability and structure analysis. NMR spectroscopy and high-resolution mass spectrometry were used to confirm the chemical structures of R91 and R89 (Figs. S3–S11). Minimal time-dependent hydrolysis of the two TSCs was observed over 48 h, strongly suggesting that the active molecules were the TSCs (Figs. S12 and S13). Moreover, structural analysis revealed that TSCs with potent activity shared specific features, such as hydroxyl and halogen groups in the first aromatic ring. Additionally, an additional halogen in the 4′ position of the second aromatic ring further enhanced, but was not required for, antimicrobial activity. Despite the relatively comparable activity of R91 and R89 against USA300 LAC, we decided to use R91 as our representative TSC in follow-up experiments given that this TSC, but not the analogs, was identified in our initial high-throughput screening.

### R91 induces copper and oxidative stress responses in *S. aureus*

**Figure 2:**
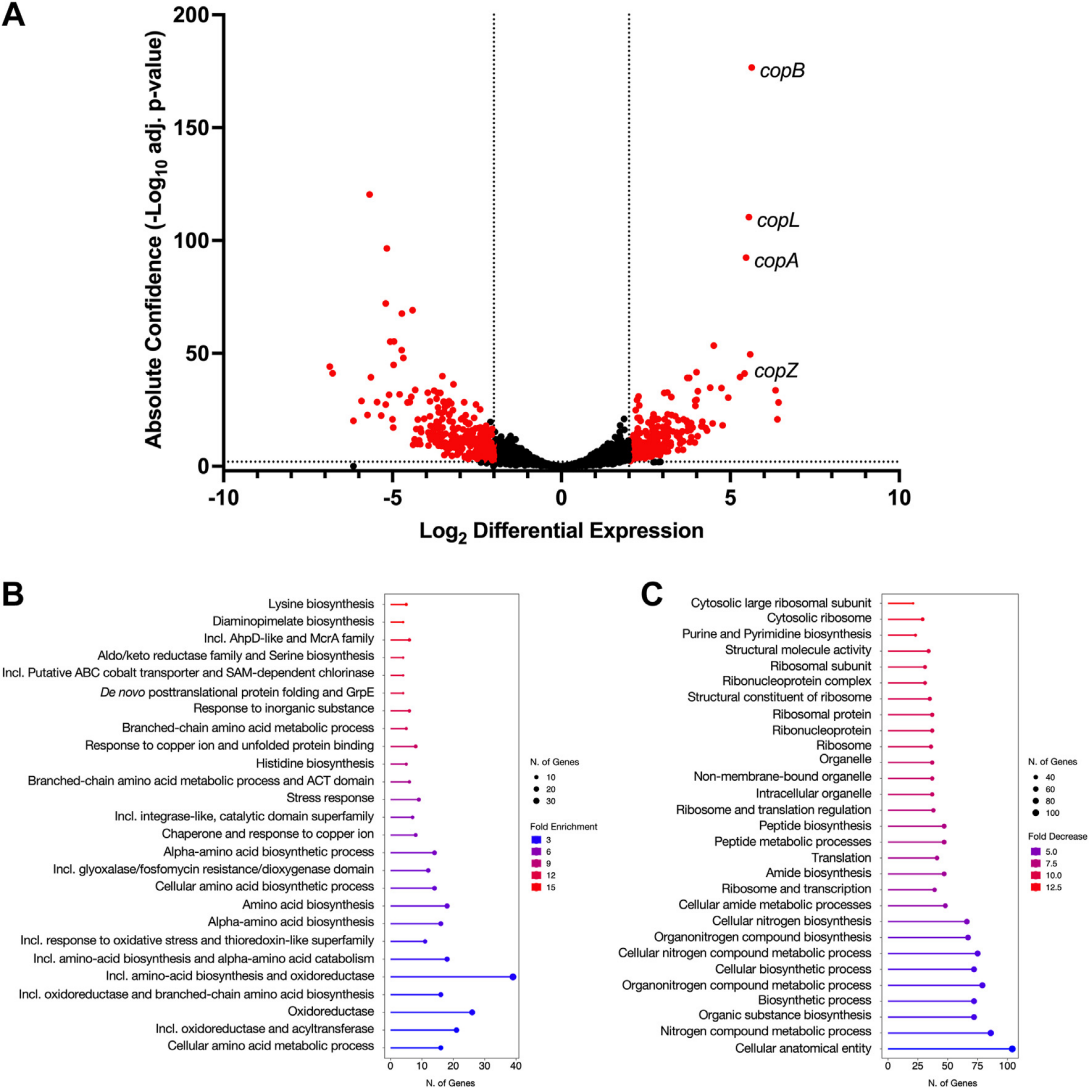
**R91 exposure results in the upregulation of copper homeostasis genes.***A*, volcano plot of RNAseq analysis of *Staphylococcus aureus* USA300 LAC in early exponential phase after 30 min exposure to sub-MIC R91 (3.125 μM). Results are from three independent experiments. Differentially expressed genes (absolute confidence >2, |log_2_ differential expression| >2) are colored in *red*. Detailed RNAseq data can be found in Table S3. Gene Ontology (GO) analysis of significantly upregulated (*B*) and downregulated (*C*) genes. Shown in (*B* and *C*) are the GO-enriched lollipop plots of differentially expressed pathways identified within the top 30 pathways. The GO terms corresponding to each pathway were initially filtered by a false discovery rate (FDR) of <0.05, before being selected by FDR and sorted for fold enrichment. The size of the dot represents the number of genes, and the color corresponds to the fold enrichment of the pathway.

RNAseq was used to gain insight into the immediate transcriptional response of *S. aureus* to TSC exposure, using R91 as the representative molecule. Bacteria in early exponential growth phase were exposed to 0.5× MIC R91 for 30 min, at which time RNA was extracted and used for RNAseq analysis. The analysis of differentially expressed genes revealed that R91 exposure elicited a dramatic transcriptional response, as 267 genes were significantly upregulated while 269 genes were significantly downregulated (Fig. 2*A*). One prominent set of genes that were significantly upregulated in response to R91 exposure were the *copA* (29-fold), *copZ* (26-fold), *copB* (32-fold), and *copL* (30-fold) genes, which are associated with copper resistance (27). This upregulation suggests that exposure of *S. aureus* to R91 is activating a response similar to a high-copper stress response (27, 28, 29), despite the fact that copper was not added to the TSB growth medium for these experiments.

Gene ontology (GO) analysis revealed several differentially regulated pathways and biological processes in response to R91 exposure (Fig. 2, *B* and *C*). Increased expression (>2-fold) of the CtsR (*clpB*, *clpC*, *ctsR*, *mcsA*, *mcsB*, *clpP*) and HrcA (*dnaK*, *hrcA*, *grpE*, *groELS*) regulons, which contain several protease and chaperone genes that are essential for resistance to heat shock and oxidative stress (30, 31), suggests activation of the stress response after R91 exposure. In particular, the upregulation of these regulons in the context of antimicrobial treatment has been associated with oxidative damage and accumulation of misfolded proteins (32). Consistent with the notion that R91 elicits an oxidative stress response, we also observed that R91 exposure induced expression of the *katA* gene (3.2-fold) encoding catalase and the *sodA* and *sodM* genes encoding superoxide dismutase (3- and 3.8-fold, respectively). Together, these results suggest a general oxidative stress response is activated upon R91 exposure.

Furthermore, R91 elicited decreased expression of pathways associated with purine and pyrimidine biosynthesis, ribosomal subunits, and protein translation genes (Fig. 2*C*), indicating induction of the stringent response (33). This shift in gene expression suggests a reallocation of bacterial resources toward stress response mechanisms over rapid growth in response to R91 exposure.

### Metal-associated antibacterial activity of R91

Our RNAseq data revealed that exposure to R91 induced the expression of copper-detoxification genes. To explore whether bacteria deficient for these genes would display altered sensitivity to the antimicrobial effects of R91, we utilized mutants lacking either *copAZ*, *copBL*, or *copAZ*, and *copBL* and evaluated the MIC of R91 on these mutant strains. Surprisingly, none of the KO strains displayed an altered MIC for R91 (Fig. S14*A*), suggesting that these genes are not required for R91 activity or resistance. Nonetheless, R91 exposure did elicit a copper stress response, potentially implying a direct interaction between R91 and copper, supported by previous studies on the metal-binding abilities of TSCs (13, 14).

To rule out the possibility that the observed effects were due to copper contamination of R91, we performed inductively coupled plasma mass spectrometry (ICP-MS) analysis. Our results confirmed that R91 was not contaminated with copper during synthesis, with the measured copper concentration being approximately 1 ppb in a 115.4 μM solution in 1% dimethylsulfoxide (DMSO), equating to an approximate molar ratio of 1:9000 (Table S4). Additionally, we verified that R89, an analog with similar activity to R91, also had no detectable copper contamination, with a similar concentration of 1 ppb in a 139.6 μM solution in 1% DMSO, also yielding a molar ratio of approximately 1:9000 (Table S4). These findings suggest that activation of copper efflux-related genes in response to R91 exposure may be a result of the drug interacting with trace copper in the media and transporting it into the bacteria and not a result of copper contamination of the molecules themselves.

**Figure 3:**
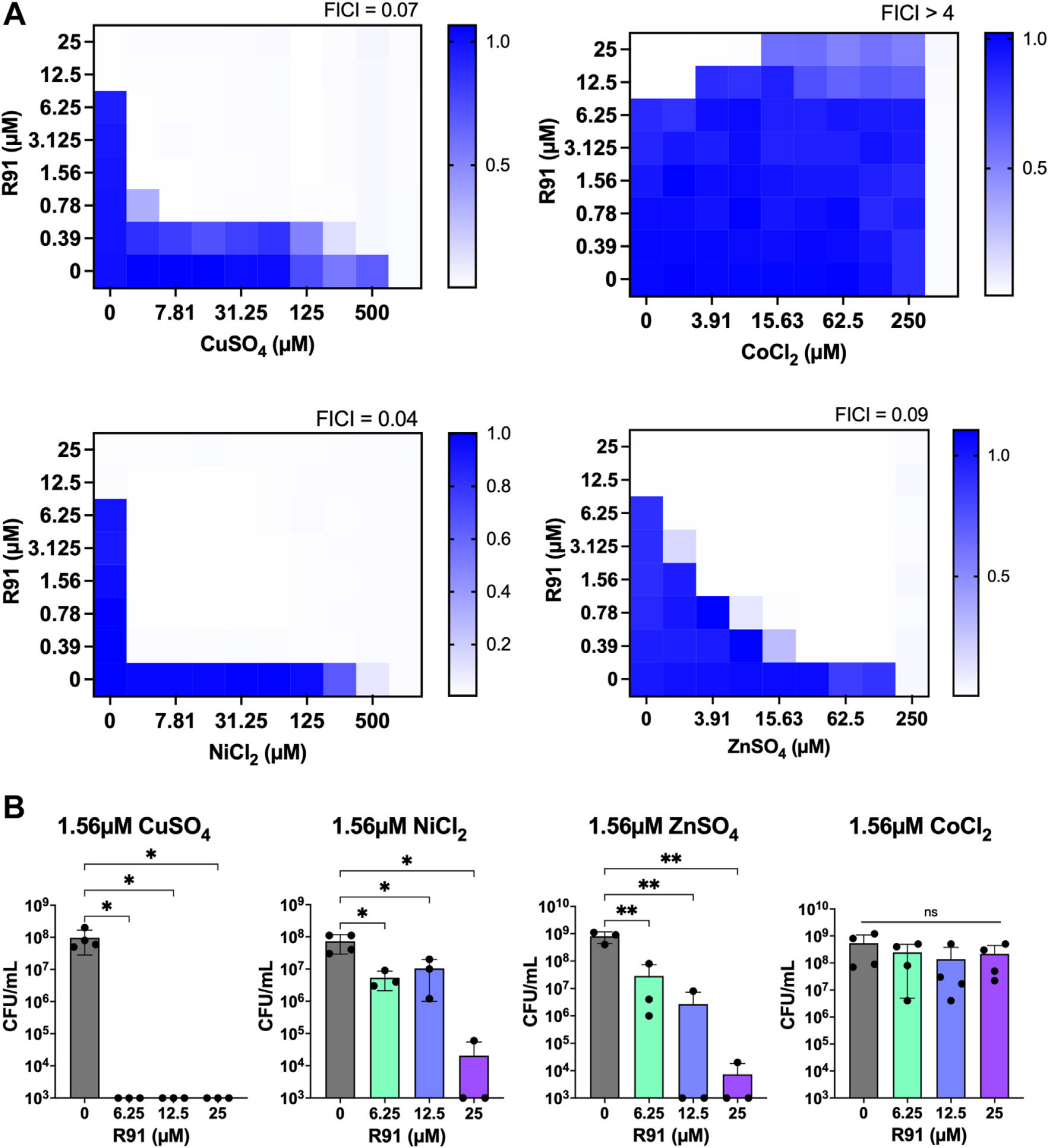
**Anti-MRSA activity of R91 is enhanced by Cu**^**2+**^**, Ni**^**2+**^**, and Zn**^**2+**^. *A*, representative checkerboard assays of R91 with CuSO_4_, ZnSO_4_, NiCl_2_, or CoCl_2_ against *Staphylococcus aureus* USA300 LAC. A diagram corresponding to the setup of the checkerboards is displayed in the report by Bellio *et al.*, 2021 (78). Growth was quantified by measuring absorbance at A_600_, and results are represented using heat maps, where a darker *blue* color corresponds to a greater bacterial density. Synergy is defined as FICI < 0.5, indifference as 0.5 < FICI ≥ 4, and antagonism as FICI > 4. The checkerboards were repeated three times independently and all replicates yielded similar results. *B*, bactericidal activity of R91 when in combination with synergistic metals. CFU/ml was quantified after bacterial exposure to R91 for 24 h. The limit of detection (LOD) was 10^3^ CFU/ml. Data points below the limit of detection (LOD) were plotted at the LOD. Data are shown as the mean ± SD from at least three biological replicates. ∗*p* ≤ 0.05, ∗∗*p* ≤ 0.01 using a one-way ANOVA with Dunnett’s multiple comparison.

To further investigate the impact of R91 and metals on *S. aureus* growth, we assessed the synergy of R91 with copper and other metals using checkerboard assays and the fractional inhibitory concentration index (FICI) to define interaction types observed. Interestingly, iron, calcium, manganese, and magnesium had little effect on the activity of R91 (Fig. S15). However, the addition of copper did result in enhanced antibacterial activity of R91, indicated by synergy (FICI ≤ 0.5) between the two (Fig. 3*A*). Similar synergistic effects were observed with nickel (FICI = 0.04) and zinc (FICI = 0.09) (Fig. 3*A*). Notably, the synergy between R91 and copper, nickel, or zinc not only reduced the MIC of R91 but also induced a bactericidal effect at much lower compound concentrations (Fig. 3*B*). Despite this, the MIC of R91 remained unchanged between various Chelex-100 treated and untreated media (Fig. S14, *B* and *C*). These findings suggest that the combined action of R91 and specific metals significantly enhances the compound's antibacterial efficacy, but that the activity of R91 is not dependent on the presence of these metals under the conditions we have tested.

Interestingly, cobalt exhibited antagonism (FICI > 4) when in combination with R91 (Fig. 3*A*). This resulted in no decrease in CFU/ml of bacteria treated with both R91 and cobalt (Fig. 3*B*). While complexation of cobalt with TSCs has been reported (34), a change in conformation or coordination of R91 molecules may result in a loss of activity or target-binding ability.

### Oxidative stress response is essential for resistance to R91

**Figure 4:**
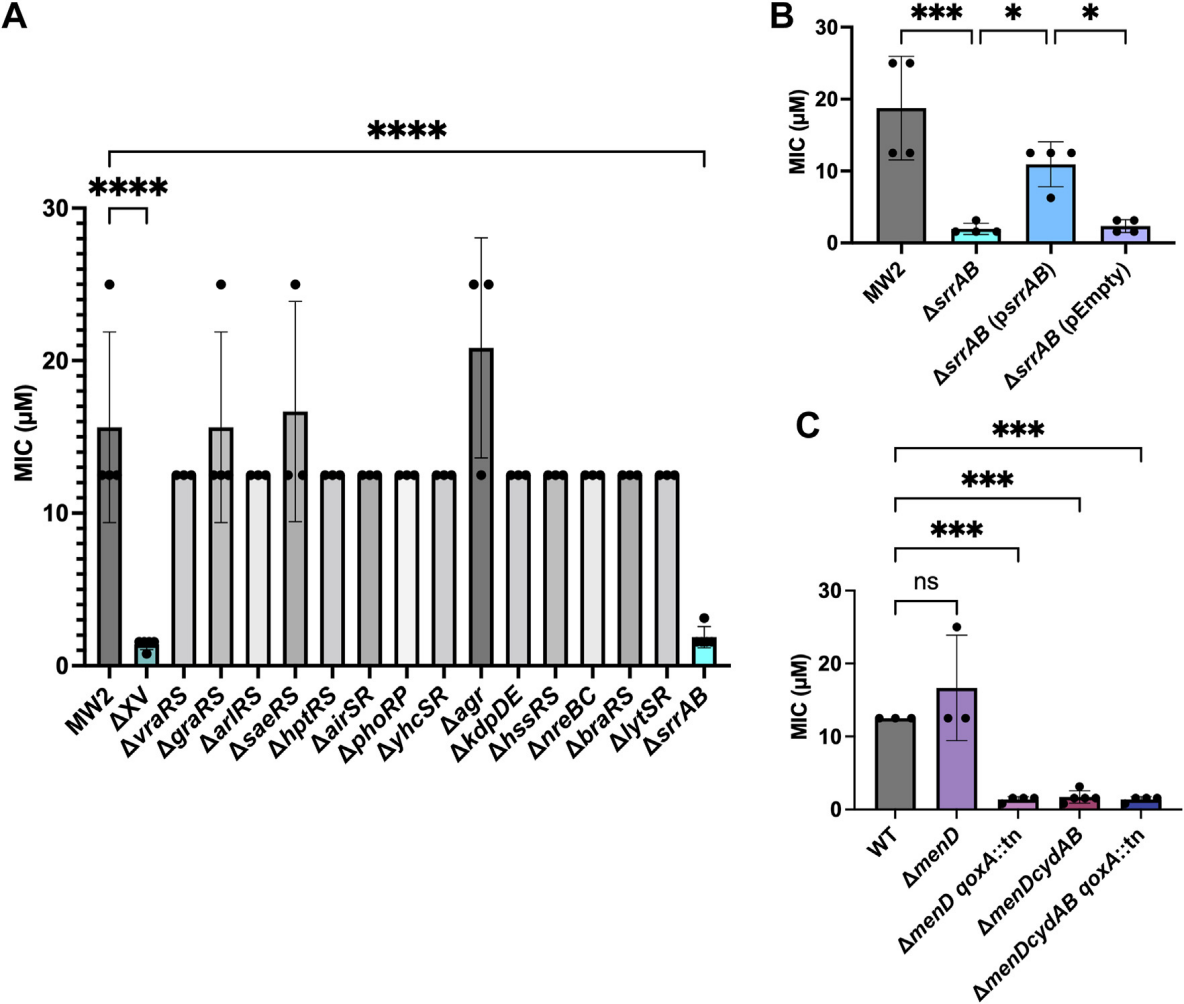
**Mutation of *srrAB* sensitizes *Staphylococcus aureus* USA400 MW2 to R91.***A*, MIC of R91 against two-component system mutants in an *S. aureus* USA400 MW2 background. Data are shown as the mean ± SD from at least three biological replicates. ∗∗∗∗*p* ≤ 0.0001 using a one-way ANOVA with Dunnett’s multiple comparisons. *B*, MIC of the *S. aureus* USA400MW2 background strain, Δ*srrAB*, complement (p*srrAB*), and empty vector (pEmpty). Data are shown as the mean ± SD from at least three biological replicates. ∗*p* ≤ 0.05, ∗∗∗*p* ≤ 0.001 using a one-way ANOVA with a Šidák multiple comparison. *C*, MIC of Δ*menD* and terminal electron acceptor mutants (*qoxA::*tn and Δ*cydAB*) in a Δ*menD* background. MIC was determined in TSB after 24 h of growth using concentrations that completely inhibited growth, as determined by measurement of A_600_. Data are shown as the mean ± SD from at least three biological replicates. ∗∗∗*p* ≤ 0.001 using a one-way ANOVA with a Šidák multiple comparison.

Two-component regulatory systems (TCRSs) enable bacteria to adapt and respond to environmental changes, including antibiotic exposure, by altering gene expression (35). Indeed, several staphylococcal TCRSs have been associated with antimicrobial resistance (36, 37). To begin to explore the role of TCRSs in resisting the antimicrobial activity of R91, we performed MIC analysis using a strain lacking all non-essential TCRSs (ΔXV) (38). This analysis revealed that ΔXV displayed heightened sensitivity to R91 as indicated by a 4-fold decrease in the MIC (Fig. 4*A*), suggesting involvement of these systems in resistance modulation. However, ΔXV did not exhibit hypersensitivity to R91 analogs, except for R31 (Fig. S16), indicating that the resistance mechanism may be specific to certain molecular structures or functional groups within the analogs.

Further MIC analyses on single TCRS mutants (38) identified heightened sensitivity in *S. aureus* lacking only the SrrAB TCRS, as indicated by the 4-fold decrease in the MIC (Fig. 4*A*). Complementation of the Δ*srrAB* mutant by providing *srrAB in trans* resulted in the loss of heightened sensitivity (Fig. 4*B*), confirming that the SrrAB two-component system is responsible for the level of R91 resistance that is observed in *S. aureus* USA400 MW2. SrrAB is an oxygen-responsive regulator involved in biofilm formation and the oxidative stress response (39, 40, 41). Thus, the hypersensitivity of the Δ*srrAB* mutant suggests that R91 may generate oxidative stress as part of its antibacterial mechanism.

In response to oxidative stress, SrrAB induces expression of the *qox* operon. In particular, *qoxA* is part of the quinol oxidase complex, contributing to respiratory electron transport (42). Therefore, we speculated that R91 may influence the oxidative stress response by interfering with the electron transport chain (ETC). CydAB, a cytochrome bd oxidase complex, and MenD, which is involved in the biosynthesis of menaquinone, are key components of the ETC and play essential roles in oxidative stress defense (43, 44). Thus, *menD*, *cydAB*, and *qoxA* mutants were used to investigate the effect of R91 on the ETC in *S. aureus*. Interestingly, while *S. aureus* deficient for *menD* did not exhibit increased sensitivity to R91, mutants deficient for *cydAB*, *qoxA* or *cydAB*, and *qoxA*, in a *menD* background were hypersusceptible to R91 (Fig. 4*C*). These data show that *S. aureus* lacking functional Qox and Cyd terminal oxidases in *menD*-deficient bacteria are hypersensitive to the antimicrobial effects of R91.

### R91 elicits hydrogen peroxide formation

**Figure 5:**
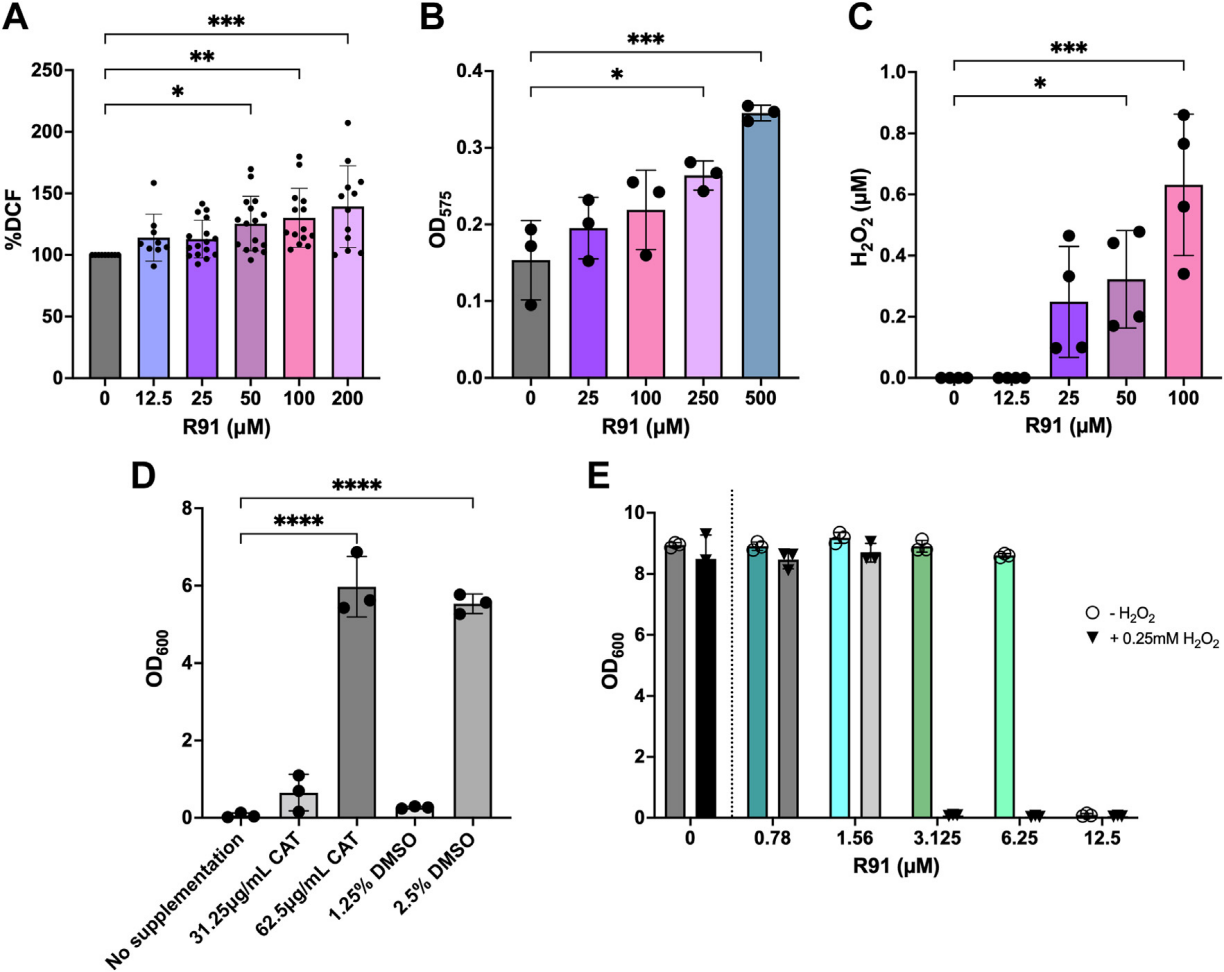
**R91 causes the production of hydrogen peroxide in *Staphylococcus aureus*.***A*, intracellular ROS levels in *S. aureus* USA300 LAC after treatment with R91. Bacteria were treated with DCFH-DA for 30 min after R91 exposure and fluorescence was measured at an excitation of 485 nm and an emission of 535 nm. Fluorescence was normalized to the A_600_ absorbances of the untreated controls. Data are shown as the mean ± SD of at least three biological replicates. ∗*p* ≤ 0.05, ∗∗*p* ≤ 0.01, ∗∗∗*p* ≤ 0.001 using a one-way ANOVA with Dunnett’s multiple comparison. *B*, intracellular superoxide (O_2_^•−^) levels in *S. aureus* USA300 LAC after exposure to R91 for 30 min. Nitro-blue tetrazolium (NBT) reduction was used to determine O_2_^•−^ levels. Data are shown as the mean ± SD of at three independent experiments. ∗*p* ≤ 0.05, ∗∗∗*p* ≤ 0.001 using a one-way ANOVA with Dunnett’s multiple comparison. *C*, H_2_O_2_ levels in *S. aureus* USA300 LAC after R91 exposure. Bacteria were incubated with R91 for 1 h, before being lysed with lysostaphin and added to Amplex red and incubated for 30 min in the dark. Fluorescence was measured at an excitation of 560 nm and an emission of 590 nm. H_2_O_2_ (μM) was quantified using an equation generated from a linear regression of a set of H_2_O_2_ standards. Data are shown as the mean ± SD of three biological replicates. ∗*p* ≤ 0.05, ∗∗∗*p* ≤ 0.001 using a one-way ANOVA with Dunnett’s multiple comparison. *D*, A_600_ of *S. aureus* treated with R91 at the MIC was determined in the presence and absence of ROS scavengers, catalase (CAT) and dimethylsulfoxide (DMSO). A_600_ was determined after exposure to R91 and scavenger for 24 h. Data are shown as the mean ± SD of three biological replicates. ∗∗∗∗*p* ≤ 0.0001 using a one-way ANOVA with Dunnett’s multiple comparison. *E*, killing of *S. aureus* USA300 LAC by R91 and R91 with sublethal H_2_O_2_. A_600_ was read after exposure to 12.5 μM R91 ± 0.25 mM H_2_O_2_ for 24 h in TSB. Data are shown as the mean ± SD of three biological replicates.

The upregulation of genes involved in oxidative stress (*i.e. katA*, *sodA*, and *sodM*) and the hypersensitivity of the aforementioned mutants to R91 prompted investigation of whether R91 induces formation of ROS. To this end, we employed dichlorodihydrofluorescein diacetate (DCFH-DA), a compound that is converted into the fluorescent product dichlorofluorescein upon exposure to ROS (Fig. 5*A*). Using this assay, we observed a significant increase in fluorescence after R91 exposure and the DCFH-DA fluorescence was directly proportional to the concentration of R91 used. Taken together, these data indicate that R91 induces ROS formation in *S. aureus*.

We next sought to identify the nature of the ROS produced in response to exposure to R91. To achieve this, we employed a nitroblue tetrazolium (NBT) assay which can detect the presence of superoxide anions (O_2_^-^) through formazan formation. This assay revealed R91 concentration-dependent formazan production but only at concentrations significantly above the MIC (Fig. 5*B*). This suggests that O_2_^-^ is likely not a major contributor to the anti-staphylococcal properties of R91.

Next, we considered whether R91 could induce the formation of hydrogen peroxide (H_2_O_2_), which can kill *S. aureus* (45). To address this, we utilized Amplex Red assays to measure H_2_O_2_ production in response to R91 treatment of *S. aureus*. In the absence of R91, the measurable level of H_2_O_2_ in *S. aureus* lysates was negligible. In contrast, in the presence of R91, a notable increase in Amplex Red fluorescence was detected (Fig. 5*C*), suggesting that R91 is causing the production of H_2_O_2_. Scavenger assays using catalase (for H_2_O_2_) and DMSO (for hydroxyl radicals) reinforced this notion as addition of either to R91-exposed cultures led to growth recovery at the MIC (Fig. 5*D*). Furthermore, indirect analysis of catalase activity using a foam formation assay (46), revealed that cultures exposed to R91 and subsequently to 30% hydrogen peroxide displayed enhanced foam formation (Fig. S17). Moreover, exposure to sub-inhibitory H_2_O_2_ concentrations (0.25 mM) reduced the MIC of R91 4-fold as compared to R91 alone (Fig. 5*E*). Finally, mutants lacking catalase (*i.e.* a *katA*::tn strain) were found to be hypersensitive to R91 and were killed at concentrations well below the bactericidal concentration of R91 against USA300 LAC (Fig. S18, *A* and *B*), while those with enhanced catalase expression (*i.e.* a *perR*::tn strain) (47) were more resistant to R91 killing, with no changes in CFU/ml at concentrations of R91 greater than 50 μM (Fig. S18*C*). Like the *perR::*tn mutant, the USA300 pKatA strain, which also overexpresses catalase, was found to be less sensitive to killing by R91, further confirming the importance of H_2_O_2_ detoxification mechanisms in R91 resistance (Fig. S18*D*). Taken together, these data establish that R91 induces ROS in the form of hydrogen peroxide and in response to R91, *S. aureus* induces the expression of active catalase enzyme that contributes to the basal level of resistance observed.

### R91 treatment can prevent and eradicate biofilm formation

**Figure 6:**
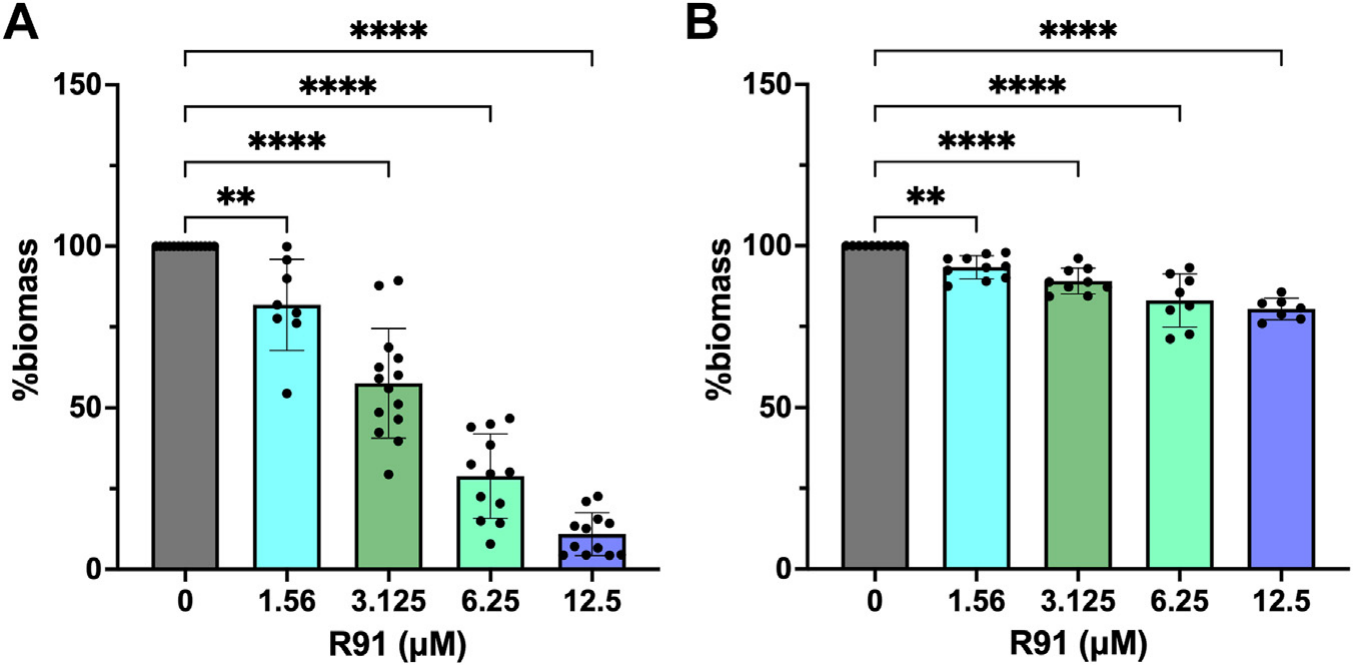
**R91 prevents and eradicates biofilms.** Biofilm formation in the presence and absence of R91 after (*A*) co-incubation of R91 and *Staphylococcus aureus* USA300 LAC for 24 h, (*B*) 24 h of R91 incubation after 24 h of initial biofilm formation. Biofilms were quantified using an A_590_ after crystal violet staining. %biomass was quantified by comparing the OD of the treated samples to the OD of the untreated samples. Data are shown as the mean ± SD of at least three independent experiments. ∗∗*p* ≤ 0.01, ∗∗∗∗*p* ≤ 0.0001 using a one-way ANOVA with Dunnett’s multiple comparison.

*S. aureus* biofilm formation is critically important in clinical settings due to its association with persistent infections and increased antibiotic resistance (48). Moreover, biofilm formation enables bacteria to evade host immunity and to resist conventional antibiotic treatments (48). We thus evaluated the prophylactic efficacy of R91 against *S. aureus* biofilm formation which involved statically culturing *S. aureus* with R91 for 24 h, followed by biofilm quantification. At a concentration of R91 4-fold below the established MIC, R91 significantly decreased biofilm formation as compared to untreated controls (Fig. 6*A*). This effect was further exacerbated at the MIC, suggesting R91 could be used to mitigate biofilm-formation in static culture.

We next investigated whether R91 could dissipate preformed biofilms. Here, biofilms were allowed to form over 24 h at which time R91 was added to the culture medium for an additional 24 h (Fig. 6*B*). These experiments revealed that at the MIC, R91 significantly reduced pre-formed biofilm. This anti-biofilm effect was also observed using R91 concentrations up to 10-fold below the MIC (Fig. 6*B*).

### Prolonged exposure to R91 does not produce TSC-resistant bacteria

To identify the R91 target(s) and to explore its mechanism of action, we next sought to identify *S. aureus* mutants that had increased resistance to R91. To this end, we screened the Nebraska Transposon Mutant Library (NTML), which is comprised of 1920 nonessential transposon mutants in an *S. aureus* USA300 background. Using this approach, we failed to identify any mutant that displayed R91 resistance. In a parallel effort, using disk diffusion assays, we attempted to isolate spontaneous resistant colonies from within zones of inhibition; however, even after 5 days of incubation, resistant colonies failed to grow within the zones of inhibition. We continued this pursuit and next performed a 14-days serial passage experiment where *S. aureus* bacteria were exposed to 0.5× MIC of R91; however, resistant colonies were never identified (Fig. S19). In contrast, USA300 LAC exposed to 0.5× MIC ciprofloxacin quickly developed resistance, with stable resistance emerging after 5 days of subculturing indicating the approach, in principle, can produce antibiotic resistant bacteria (Fig. S19). Next, these experiments were extended out to 50 days, and despite this prolonged exposure to sub-MIC R91, resistance was never observed. Attempts to induce resistance in the presence of copper and with the copper efflux-deficient mutant Δ*copAZB* also failed to produce resistant colonies. Taken together, these data indicate that resistance to R91 in *S. aureus* does not readily occur, at least under the conditions employed here.

### R91 can kill *S. aureus* residing within macrophages

**Figure 7:**
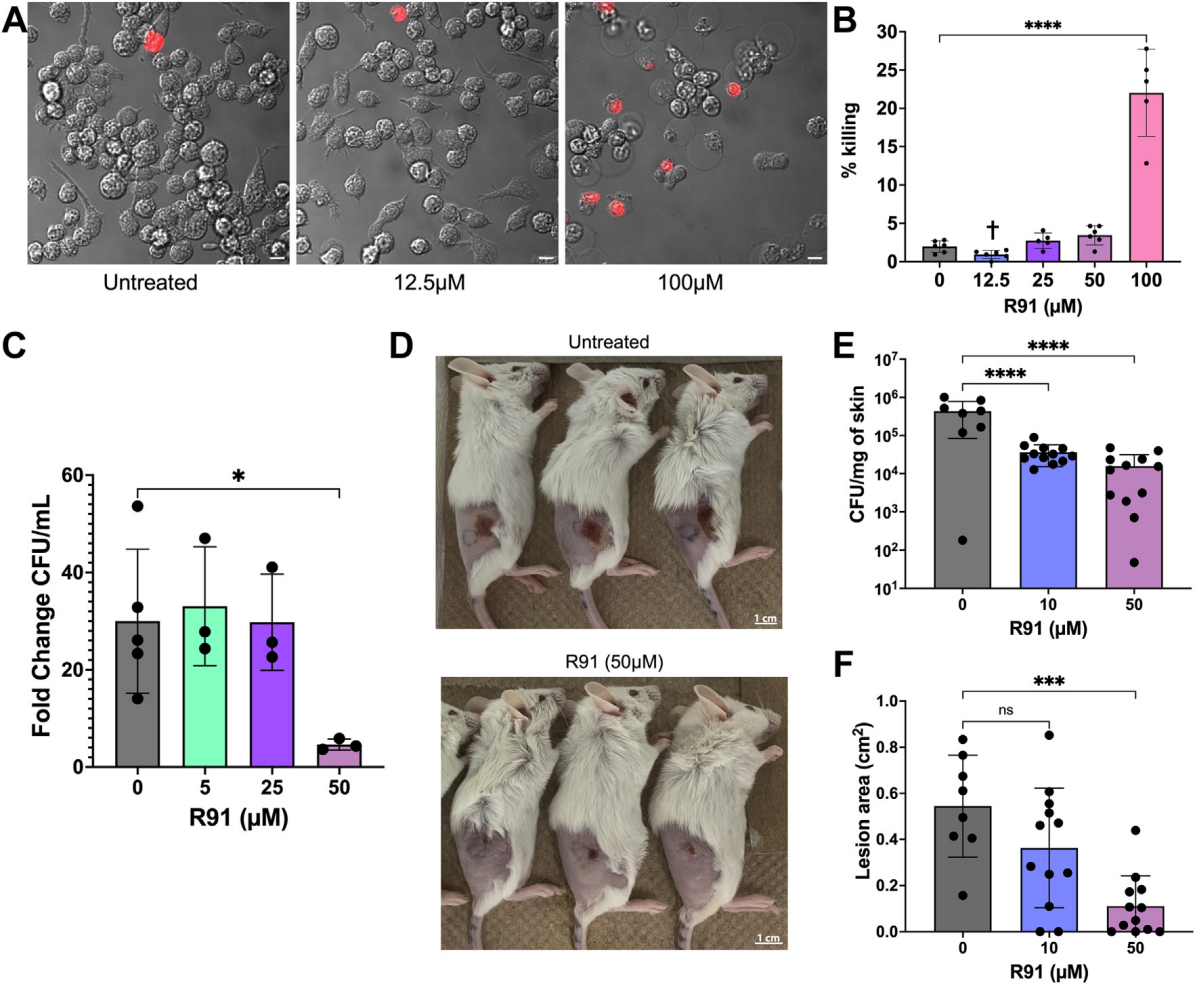
**R91 is noncytotoxic and abrogates infections in macrophages and in a skin infection model.** R91 was incubated with RAW 264.7 macrophages in RPMI supplemented with 5% fetal bovine serum (FBS). The MIC of R91 in the RPMI for *Staphylococcus aureus* USA300 LAC is 6.25 μM. *A*, after 24 h, the cells were stained with propidium iodide (PI) and imaged using a widefield microscope at 63× magnification with all scale bars representing 10 μm (*B*) and the %killing of macrophages was calculated using the number of PI-stained cells compared to the total number of cells. The MIC (μM) of R91 against USA300 LAC is indicated by †. Data are shown as the mean ± SD of at least five independent experiments ∗∗∗∗*p* ≤ 0.0001 using a one-way ANOVA with Dunnett’s multiple comparison. *C*, R91 was added to *S. aureus*–infected macrophages 1.5 hpi. The cells were then lysed at 12 hpi and CFUs were quantified to determine the ability of R91 to inhibit bacterial replication intracellularly. Data are shown as the mean ± SD of three independent experiments. ∗*p* ≤ 0.05 using a one-way ANOVA with Dunnett’s multiple comparison. *D*, representative images showing skin lesions 3 dpi. Mice were subcutaneously co-injected with *S. aureus* USA300 LAC and R91. An additional dose of R91 was given at 1 dpi. The mice were sacrificed at 3 dpi and (*E*) CFU/mg of homogenized lesion and (*F*) lesion sizes were quantified. Data are shown as the mean ± SD of three independent experiments. ∗∗∗*p* ≤ 0.001, ∗∗∗∗*p* ≤ 0.0001 using a one-way ANOVA with Dunnett’s multiple comparison.

Several TSCs are currently approved for anti-cancer and anti-Mtb treatments (49, 50). Despite this, their use is often impeded by poor water solubility, leading to challenges in bioavailability and potential toxicity (51, 52). To investigate whether R91 was noncytotoxic, we conducted experiments using RAW 264.7 macrophages as a model eukaryotic immune cell. Macrophages were either exposed to R91 or treated with an equivalent volume of DMSO for 24 h and then stained with propidium iodide (PI) to evaluate cell death (Fig. 7*A*). Quantification of dead cells through PI staining (Fig. 7*B*) revealed that R91 demonstrated noncytotoxic behavior at concentrations up to 4× greater than the MIC of the compound for *S. aureus*. However, at 100 μM (8× the MIC), cytotoxic effects were observed. Importantly, most R91 analogs were found to be cytotoxic at the concentration required to inhibit USA300 LAC (Fig. S20). While R89 was an exception to this, cytotoxicity occurred at levels notably below that of R91, and this analog was found to be cytotoxic at concentrations required to inhibit several non-USA300 strains of *S. aureus*, as well as other Gram positive bacteria.

*S. aureus* exhibits the ability to survive and reproduce within both murine and human macrophages (53, 54, 55, 56, 57, 58). The ability to treat these intracellular reservoirs is an attractive feature of novel anti-staphylococcal agents. Thus, RAW 264.7 macrophages already infected with USA300 LAC were treated with up to 50 μM R91, which was already established to be noncytotoxic towards macrophages. These experiments revealed that akin to untreated macrophages, infected macrophages treated with R91 below 50 μM failed to restrict *S. aureus* growth. In contrast, at 50 μM R91, the ability of the bacteria to grow inside macrophages was significantly impaired as indicated by the reduced fold change in CFU/ml at 12hpi as compared to the other conditions (Fig. 7*C*). These data indicate that R91 can antagonize *S. aureus* growth from within the model macrophage.

### R91 reduces lesion size and bacterial burden in a skin infection model

As a first step towards determining whether the anti-MRSA efficacy of R91 could be observed *in vivo*, we conducted murine SQ skin infection experiments using *S. aureus* USA300 LAC. This strain is a leading cause of skin and soft tissue infections in humans and is widely used within the research community for its ability to cause SQ infections in mice. Here, a mixture of USA300 LAC and R91 (mixed immediately prior to SQ injection) or untreated bacteria alone were injected SQ into the flanks of mice that had been depilated 24 h prior to infection (Fig. 7*D*). At 72 h post-infection, lesion sizes and the bacterial burden for individual lesions were measured. These experiments revealed that R91 caused significant reduction in both bacterial burden (Fig. 7*E*) and in lesion size (Fig. 7*F*) as compared to untreated animals in this model.

## Discussion

MRSA is a high-priority pathogen of concern due to its ability to both acquire and upregulate antimicrobial resistance genes. The escalating incidence of MDR-MRSA emphasizes the urgent need for the discovery of novel antibiotics. TSCs, with their unique and pleiotropic mechanisms of action, hold promise in addressing the challenges posed by the increasing prevalence of MDR-MRSA. Here, using a high-throughput screen, we aimed to identify novel antimicrobials that were effective against MRSA and disrupted metal homeostasis. A number of antimicrobials that act through the modulation of *S. aureus* metal homeostasis have been previously reported (23, 24, 25, 26). We therefore chose to focus specifically on TSCs because they not only interact with metals to enhance metal-related toxicity (59) but also exhibit high potency against *S. aureus* and target a variety of cellular processes, making them promising candidates for novel antimicrobial strategies (9, 10, 11, 12, 13, 16, 19, 60). Nevertheless, further investigation is warranted to elucidate the full scope of their antimicrobial activity and potential application in combating MRSA infection.

In our high-throughput screen of the Maybridge bioactives library, we identified a TSC with an unexplored scaffold and high efficacy, “R91” (Fig. 1*A*), and using this compound and related analogs, we have begun to characterize their activity. Similar to R91, analogs R89, R29, R39, and D79 were effective against all Gram-positive bacteria tested, with the exception of *S. saprophyticus* (Fig. 1*B*). Conceivably, given that R91 induces H_2_O_2_ production (Fig. 5), the observed *S. saprophyticus* resistance is likely due to the fact it carries a second catalase gene in its genome that could endow this species with an enhanced ability to detoxify H_2_O_2_ (61). Notably, while analogs R31 and R10 were found to be ineffective against most Gram-positive bacteria and all Gram-negative bacteria tested, R31 was capable of inhibiting *S. chromogenes*, *S. pyogenes, S. agalactiae*, and *M. luteus*. Collectively, this suggests that TSCs as a class of antimicrobials can have an extensive range of activity and indicates that particular TSCs could be used to address distinct bacterial species and strains. The efficacy of these analogs against various staphylococcal species allowed for the identification of key structural moieties that appear to be associated with antimicrobial activity. Indeed, the presence of a halogen and a hydroxyl group on the first aromatic group is required for TSC antimicrobial function, indicating these are likely required for target binding. Phenol moieties within TSCs have been predicted to facilitate interactions with copper (62). Furthermore, we observed increased activity with the presence of an additional halogen group, as has been shown previously (10, 63). As halogenated antimicrobials are known for their enhanced lipophilicity and ability to cross the cellular membrane (64), it is possible that TSCs possessing additional halogens have enhanced antimicrobial activity due to increased intracellular concentrations.

While the specific cellular target for R91 is not known, we provide compelling evidence showing that trace metals are intertwined with its activity. Indeed, the RNAseq data (Fig. 2*A*) demonstrate that genes, *copAZ* and *copBL*, involved in copper detoxification are induced upon *S. aureus* exposure to R91, despite the fact that we observed activity of R91 without the addition of exogenous copper and in metal-deplete media (Figs. 1*A* and S14*B*). Why R91 induces these copper-specific genes is presently unclear, but conceivably, if R91 directly binds copper, entry of the drug into the cell with trace copper from the media may cause a rapid increase in intracellular copper. Regardless, there exists an important relationship between R91 and copper as evidenced by the fact that copper and the TSC synergize to produce enhanced anti-staphylococcal activity (Fig. 3).

Clearly, the effects of R91 on the cell are complex as evidenced by the differential expression of genes linked to general stress responses and altered biosynthetic pathways, in addition to copper intoxication. Indeed, transcriptomic analysis (Fig. 2, *B* and *C*) reveals a stringent response in *S. aureus* triggered by R91, with downregulation of nucleotide biosynthesis and protein translation pathways possibly indicating a trade-off between growth and stress responses (33). The stringent response is also characterized by an upregulation in branched-chain amino acid biosynthesis and genes related to oxidative stress resistance (33), and upregulation of genes involved in both processes is observed after acute R91 exposure. Furthermore, the transcriptomic response of *S. aureus* to R91 closely mirrors the response of the pathogen to lapachol, a naphthoquinone antibiotic that induces a quinone and oxidative stress response in *S. aureus* (65). As with lapachol, R91 strongly induced expression of the CtsR, HrcA, PerR, NsrR, MhqR, and QsrK operons. Specifically, the PerR regulon responds to hydrogen peroxide, leading to the de-repression of genes involved in the oxidative stress response (66). This suggests R91 may induce oxidative stress and protein damage, as indicated by the heightened expression of genes controlling protein quality control machinery (*ctsR* and *hrcA*).

Consistent with the notion that R91 induces oxidative stress, *S. aureus* bacteria deficient for *srrAB*, *qoxA*, and *cydAB* all display hypersensitivity to R91 (Fig. 4). Previous studies have demonstrated that strains lacking the SrrAB two-component system are hypersensitive to H_2_O_2_-induced killing (41). Furthermore, SrrAB co-regulates expression of genes involved in the response to oxidative stress, including *katA*, *ahpC*, and *dps* (40, 67). This activation of oxidative stress response genes is linked to the ability of SrrAB to sense impaired electron flow and correspondingly modulate expression of the *qoxABCD* and *cydAB* genes required for cytochrome biosynthesis and assembly (39).

Excessive ROS production can result in oxidative stress, causing damage to cellular components such as proteins, lipids, and DNA (59). ROS production has been associated with the bactericidal effects of certain antibiotics (68, 69). As such, we believe our data support the notion that ROS production is a supplementary mechanism of R91 that is enhanced in the presence of copper or at elevated concentrations of R91. Previously, copper complexes and other antimicrobials have been shown to specifically induce H_2_O_2_ production in bacterial cells (68, 70). Given the RNAseq results, as well as the hypersensitivity of mutants deficient in their response to oxidative stress, we postulated that R91 might cause the generation of H_2_O_2_. In support of this, using both direct and indirect methods, we provide compelling evidence showing that R91 induces ROS in the form of H_2_O_2_ and conceivably H_2_O_2_ contributes to the antimicrobial activity of this TSC (Fig. 5).

Biofilms, a key virulence mechanism employed by bacteria like *S. aureus*, pose significant challenges clinically due to their ability to shield bacteria from antimicrobials and evade the immune system, leading to chronic infections and treatment failures (39, 41, 48). Notably, R91 was found to be capable of preventing biofilm formation, as well as disrupting established biofilms, even at concentrations 4-fold below the established MIC (Fig. 6).

The two-component system SrrAB plays a role in biofilm formation by regulating the *ica* operon, which is responsible for polysaccharide intracellular adhesin production (41). Additionally, copper and ROS antagonize biofilm formation by repressing genes essential for biofilm development, such as *sae*, *agr*, and *eap* (28, 71). The anti-biofilm effect of R91, coupled with the H_2_O_2_ production caused by this TSC, aligns with the ROS-linked effect of other antibiotics on biofilm formation (72, 73). The mechanism behind the action of R91 on biofilms may involve H_2_O_2_ production or direct targeting of SrrAB, warranting further investigation into this TSC’s role in resisting R91.

The multifaceted mechanism of action exhibited by R91 is likely a key factor preventing the development of resistance by MRSA. *S. aureus* is well-known for its propensity to develop resistance to antibiotics (74). Therefore, it is crucial that new antimicrobials are designed with mechanisms that minimize the likelihood of selecting for mutants displaying resistance. Remarkably, even after up to 50 days of serial passage in the presence of R91, both with and without copper, *S. aureus* bacteria displaying heightened resistance to R91 could not be acquired (Fig. S19). These findings underscore the potential for R91 as an effective treatment option.

Encouraged by the lack of resistance development, we proceeded to assess the clinical efficacy of R91 by evaluating its ability to clear intracellular infections and to treat murine skin infection. *S. aureus* is known to reside, replicate, and persist within macrophages, leading to chronic infections (53). Thus, it is essential for new antibiotics targeting *S. aureus* to be capable of entering the macrophage and targeting these intracellular reservoirs. R91 demonstrated efficacy against intracellular macrophage infections, albeit only at levels above the MIC, but still below cytotoxic concentrations (Fig. 7*C*). The utility of R91 to treat SQ skin infections *in vivo* was also assessed and revealed that prophylactic R91 treatment effectively reduced the bacterial burden within skin lesions and reduced lesion size (Fig. 7, *E* and *F*). Future experiments will focus on the ability of this TSC in ameliorating other types of infection and investigate the post-infection treatment efficacy of this compound.

Together, our data demonstrate the potent efficacy of the TSC R91 against MDR MRSA, revealing a multifaceted mechanism of action that includes prevention of biofilm formation and induction of reactive oxygen species production. The lack of resistance development to R91 and its effectiveness in treating intracellular infections and skin lesions underscore its potential as a versatile and promising antimicrobial agent for combating antibiotic-resistant *S. aureus* infections.

## Experimental procedures

### Bacterial strains and plasmids

Bacterial strains and plasmids are listed in Table S1. All strains were cultured at 37 °C with shaking at 200 rpm, except for *Streptococcal* species which were grown without shaking. All Staphylococcal species and strains, as well as most Gram-positive species were grown in TSB or on TSA plates, unless otherwise indicated. Streptococcal species were grown in Todd Hewitt Broth supplemented with 1% yeast extract (THY) and on THY agar plates. *Escherichia coli* and *Pseudomonas aeruginosa* were grown in LB or on LB agar.

### High-throughput screen

*S. aureus* strain USA300 LAC was inoculated into either Chelex 100–treated CDM-G (chemically-defined medium with 0.4% glucose) (75) or CDM-G with 30 μM FeSO_4_ in 96-well plates to a final A_600_ of 0.001. These plates contained 20 μM of each of the 936 compounds sourced from MayBridge that were previously identified as bioactives against MSSA (20). The plates were then incubated with shaking for 24 h at 37 °C followed by A_600_ determination. The screens followed general screening protocols that have been described previously (76). The primary hits were then confirmed for activity using broth dilution in Chelex 100–treated CDM-G then TSB using *S. aureus* USA300 LAC.

### Determination of MIC

Bacteria were washed and normalized to an A_600_ of 1 before being inoculated into TSB, LB, or THY containing various concentrations of TSC (dissolved in DMSO) to a final A_600_ of 0.01. Cultures were grown for 24 h at 37 °C with shaking, before the A_600_ was measured. The exception to this was *S. pyogenes* and *S. agalactiae*, which were grown without shaking. The concentration of TSC that completely inhibited growth was determined to be the MIC.

### Concentration-dependent bactericidal effect of R91

An overnight culture of *S. aureus* was inoculated 1:100 into PBS or MHB containing R91 and/or metal at concentrations ranging from 6.25 μM to 200 μM. After incubation for 24 h at 37 °C, 10 μl drops of a serial dilution were plated onto TSA. The plates were then incubated for an additional 24 h and CFU/ml were determined.

### Chemical characterization of R91 and R89

NMR spectra were obtained in D_2_O or D_2_O/DMSO-*d*_*6*_, using a 600 MHz Bruker Ascend 600 instrument. NMR chemical shifts are reported in ppm and are calibrated against the residual solvent signals of DMSO-*d*_*6*_ (2.5 ppm). Coupling constants (*J*) are given in Hz. Electrospray ionization mass spectrometry was performed using a Bruker microOTOF 11 spectrometer operating in negative ion mode.

#### R91

^1^H NMR (600 MHz, DMSO-d6) δ 11.97 (s, 1H), 10.14 (br s, 1H), 10.08 (s, 1H), 8.44 (s, 1H), 8.20 (s, 1H), 7.55 (d, J = 2.6 Hz, 1H), 7.53 (d, J = 2.4 Hz, 1H), 7.42 (dd, J = 8.4, 2.4 Hz, 1H), 7.20 (d, J = 8.4 Hz, 1H), 2.22 (s, 3H). ^13^C NMR (600 MHz, DMSO-d6) δ 178.4, 151.2, 139.2, 138.4, 138.1, 133.2, 132.9, 131.6, 130.3, 129.3, 125.5, 124.7, 123.2, 120.0, 18.0. HRMS: calcd [M]- (C15H12BrCl2N3OS):430.9261 Found (ESI): 430.9136

#### R89

^1^H NMR (600 MHz, DMSO-d6) δ 11.94 (s, 1H), 10.24 (s, 1H), 10.16 (br s, 1H), 8.45 (s, 1H), 8.19 (s, 1H), 7.55 (d, J = 15.7 Hz, 1H), 7.52–7.47 (m, 2H), 7.25–7.18 (m, 2H). ^13^C NMR (600 MHz, DMSO-d6) δ 177.2, 160.3 (d, J = 241 Hz), 151.22, 138.8, 135.90, 135.89, 130.4, 129.1, 125.7, 124.6, 123.11, 115.3 (d, J = 23 Hz). 19F NMR (600 MHz, DMSO-d6) −116.84 (tt, J = 9.0, 5.1 Hz). HRMS: calcd [M]- (C14H10Cl2FN3OS): 356.9905 Found (ESI): 356.9911.

### Stability test of R91 and R89

R91 (5 mg) and R89 (5 mg) were each dissolved in 0.6 ml of 5:1 phosphate buffered D_2_O (0.01 M, pH 7.4):DMSO-*d*_*6*_ (5:1). The solutions were incubated at 37 °C and ^1^H NMR spectra were recorded at various time points over 48 h.

### RNAseq and GO analysis

An overnight culture of USA300 LAC was inoculated 1:100 into TSB and grown to an A_600_ of 1.5 at 37 °C with shaking. 3.125 μM of R91 or an equal volume of DMSO was added to the treated cultures and all cultures were then grown for an additional 30 min. The bacteria were then normalized to an A_600_ of 3, pelleted, and treated with RNA-protect before freezing at −80 °C overnight. RNA was extracted using the QIAGEN RNAEasy kit. Briefly, cell pellets were thawed in 750 μl TE buffer (pH 8), before being resuspended in 500 μg/ml of lysostaphin. The pellets were then incubated at 37 °C for 1 h. RLT buffer and ethanol were added, and the solution was added to the RNeasy mini spin column to extract RNA according to the manufacturer’s protocol. DNAse treatment was performed using the TURBO DNA Free kit (Invitrogen) for 2 × 30 min, followed by inactivation with the supplied DNase Inactivation Reagent. Cleaned RNA was stored in sodium acetate and ethanol at −20 °C overnight. RNA sequencing was performed by the Microbial Genome Sequencing Center in Pittsburgh. Data analysis, including read mapping and differential expression, were performed by the Microbial Genome Sequencing Center in Pittsburgh. Data analysis was conducted using the Geneious Prime software package.

GO analysis was conducted using ShinyGO (77). Differentially expressed genes (absolute confidence >2, log_2_ differential expression >2) were sorted by a false discovery rate of <0.05 before being selected by FDR and sorted for fold enrichment.

### Metal-associated activity and dependence of R91

The synergy of R91 with metals was determined by checkerboard assays as previously reported (78). A clear diagram of checkerboard organization and set up can be found in the above citation. Briefly, R91 and various metals were serially diluted down each axis of a 96-well plate. The wells were then inoculated with USA300 LAC to obtain a final A_600_ of 0.01. The plates were subsequently incubated for 24 h at 37 °C with shaking. The FICIs were calculated using the following formula:

FICI= (MIC_Drug__A_ in combination/MIC_Drug__A_ alone) + (MIC_Drug_ _B_ in combination/MIC_Drug__B_ alone)

where synergy is defined as a FICI ≤ 0.5, indifference as 0.5 <FICI ≤ 4, and antagonism is defined as FICI >4.

## ICP-MS of compounds R91 and R89

ICP-MS analysis was carried out in the Louisiana Animal Disease Diagnostic Laboratory. Briefly, R89 and R91 were dissolved to 5 mg/ml in DMSO. Each compound was diluted 100× into Ultrapure water (VWR International) with 2% trace metal grade HNO_3_ (VWR) in triplicate. To control for DMSO metal content, a vehicle control consisting of 1% DMSO and 2% trace metal grade HNO_3_ in Ultrapure water was analyzed in triplicate. Hundred microliters of each sample was analyzed for ^24^ Mg, ^43^Ca, ^55^Mn, ^59^Co, and ^63^Cu on a PerkinElmer NexION 2000 interfaced with a PerkinElmer Micro DX autosampler (PerkinElmer) running Syngistix Software version 3.3 (PerkinElmer). Instrument voltages were optimized empirically through a tune just prior to analyzing the samples. Quantification was performed by comparing the counts per second of each analyte to an external calibration standard (VWR) containing 1000, 100, 10, or 1 ppb of each metal.

### PCR and construct generation

Genomic DNA was isolated from *S. aureus* USA400 MW2 (for *srrAB*) and *S. aureus* USA300 LAC (for *katA*) by phenol-chloroform extraction. Primers containing a *KpnI* or *SacI* restriction site were used to amplify *srrAB* and *katA* (see Table S2 for primers). The amplified product was ligated into pALC2073 that was also digested using *KpnI* and *SacI*. The ligated product was transformed into *E. coli* DH5α. Plasmids isolated from *E. coli* were then passaged through *S. aureus* RN4220, and subsequent plasmids isolated from this strain were introduced into electrocompetent USA400 Δ*srrAB* or USA300 LAC.

Markerless deletions of *menD* and *cydAB* were constructed using the pKOR1 system, as previously described (79). Briefly, upstream and downstream regions flanking the *menD* and *cydAB* regions were PCR amplified (see Table S2 for primers) and recombined into pKOR1. The resulting vectors were then passaged through *S. aureus* RN4220 before being introduced into electrocompetent USA300 LAC. Allelic replacement of *menD* and *cydAB* was confirmed using PCR and DNA sequencing. The *qoxA*::tn mutant was taken from the NTML library and mobilized using standard phage transduction. Phage lysate was prepared from the donor strain using phage 80α and recipient strains (Δ*menD* and Δ*menD* Δ*cydAB*) were infected. Transductants were selected for using erythromycin and confirmed using PCR.

### Detection of ROS formed by R91 treatment of *S. aureus*

#### Dichlorodihydrofluorescein diacetate

DCFH-DA was used to look at the production of general reactive oxygen species using previously described methods, with modifications (73). USA300 LAC was subcultured to an A_600_ of 1 and grown for 1 h at 37 °C with shaking. The culture was split into 1 ml aliquots and R91 was added at various concentrations. The cultures were then grown for an additional 2 h. The bacteria were then washed twice with PBS and normalized to an A_600_ of 1 in 500 μl of PBS. The solutions were then diluted 1:10 and 135 μl of each diluted culture was plated in a 96-well plate. Twenty microliters of 100 μM DCFH-DA was added to each well and the plate was incubated at 37 °C for 30 min, protected from light. Fluorescence was read at an excitation of 485 nm and emission of 535 nm and corrected for background fluorescence of an uninoculated control. General ROS production was quantified by normalizing the fluorescence of treated cells to untreated cells.

#### Amplex Red

The Amplex Red Hydrogen Peroxide/Peroxidase Assay Kit from Thermo Fisher Scientific was used to detect H_2_O_2_ production after R91 exposure according to the manufacturer’s protocol and previously established methods with modifications (68). USA300 LAC was grown to early exponential phase (A_600_ ∼ 1.5) before R91 at various concentrations was added for 1 h. Bacteria were washed and normalized to an A600 of 1 in PBS and the pellet was resuspended in 50 μl of lysostaphin (0.01 μg/μl) for 30 min at 37 °C to lyse the cells. Fifty microliters of Amplex Red working solution, prepared according to the manufacturer’s instructions, was added to the lysed cells and incubated at room-temperature for 30 min, protected from light. The H_2_O_2_ standard curve was prepared according to the manufacturer’s instructions. Fluorescence was read at an excitation of 545 nm and an emission of 590 nm, and background fluorescence was corrected for using an untreated control. H_2_O_2_ produced after R91 exposure was determined by comparing background-corrected fluorescence of treated samples to the H_2_O_2_ standard curve generated.

#### Nitroblue tetrazolium

Nitroblue tetrazolium (NBT) assays were performed as previously described (80). Briefly, various concentrations of R91 were added to 0.1 ml of USA300 LAC at an A_600_ of 0.1 in Hank’s balanced salt solution. 0.5 ml of NBT (1 mg/ml) was added and the solutions were incubated at 37 °C for 30 min. 0.1 ml of 0.1 M HCl was added, and the tubes were centrifuged at 1500*g* for 10 min. Pellets were treated with 0.6 ml DMSO to extract the reduced NBT (formazan) product. 0.8 ml of PBS was added and the absorbance was read at 575 nm to quantify intracellular superoxide production.

#### Hydrogen peroxide and scavenger supplementation

MICs were performed as described above, with the addition of 0.25 mM H_2_O_2_, and various concentrations of catalase and DMSO to either synergize or antagonize the activity of R91.

#### Foam-based determination of catalase activity

Catalase activity was assessed visually using foam height, as previously described (81), with modifications. USA300 LAC was grown to early exponential phase (A_600_ = 1.5) before R91 or hydrogen peroxide at various concentrations were added, and the bacteria were grown for an additional hour. Cells were normalized to an A_600_ of 2 in 1 ml of PBS after incubation. 100 μL of 1× Triton-X (in PBS) and 100 μL of 30% hydrogen peroxide were added and mixed thoroughly. The tubes were then incubated at room temperature for 15 min to allow the reaction to complete. Foam height was measured using a ruler and normalized to the level of foam produced by an untreated control.

### Screening assays for resistance to R91

#### Evolutionary adaptation

Overnight cultures of *S. aureus* were inoculated to an A_600_ of 0.01 in fresh TSB in the presence and absence of either 0.5× MIC R91 (6.25 μM) or ciprofloxacin (25 μM). After 24 h of incubation, the cultures were again subcultured in the presence and absence of R91 or CIP, and this process was repeated for up to 50 days. MICs were taken from days 0 to 14 to determine resistance development in both the R91-exposed and CIP-exposed cultures. The concentration of CIP was increased as resistance development was observed. 50 μM of copper was added where indicated.

#### Disk diffusion

Overnight cultures of *S. aureus* were normalized to an A_600_ of 1. Sterile cotton swabs were used to spread plate this culture onto TSA or Mueller-Hinton agar. Sterile paper disks were infused with 10 μL of R91 with various concentrations and allowed to dry before being placed on top of the bacteria. Plates were then incubated for up to 72 h at 37 °C.

#### NTML screen

The NTML library is composed of 1920 nonessential transposon mutants in an *S. aureus* USA300 background (82). Transposon mutants were inoculated into TSB in 96 well plates from frozen stock cultures. The mutants were then grown at 37 °C for 24 h with shaking. Mutants were then subcultured 1:100 into 96 well plates with fresh TSB containing R91 at either 1.56 μM or 50 μM to screen for hypersusceptible (decreased MIC) or resistant (increased MIC). The plates were then incubated for 24 h at 37 °C with shaking. Growth was determined spectrophotometrically at A_600_.

### Biofilm assays

Biofilm assays were performed as previously described (83) with modifications. Two hundred microliters of TSB, supplemented with 0.4% w/v glucose, was inoculated in triplicate with a 1:100 dilution of an overnight culture of USA300 LAC into a 96-well plate. For pre-treatment, various concentrations of R91 were co-incubated with the bacteria. The plate was then statically incubated at 37 °C for 24 h. For pre-formed biofilms, the above is followed, without the addition of R91. After 24 h, the supernatant was replaced with fresh TSB + 0.4% glucose containing various concentrations of R91. The plates were then incubated for an additional 24 h. Following incubation, the supernatant was removed, and the biofilms were washed three times with PBS. Biofilms were then fixed by drying the plate inverted at 42 °C for 1 h. Subsequently, crystal violet (0.4% w/v) was used to stain the cells for 15 min. Excess stain was removed by washing with distilled H_2_O twice. The remaining crystal violet was dissolved in glacial acetic acid (10% v/v), and the absorbance was measured at 595 nm. Absorbance values were normalized by subtracting the background absorbance of untreated/inoculated wells and further normalized using the absorbance of the untreated USA300 LAC strain, setting it to 1 or representing 100% growth.

### R91 cytotoxicity assays

RAW 264.7 macrophages were re-seeded into flasks in RPMI media supplemented with 5% fetal bovine serum. The media contained either DMSO (vehicle control) or various concentration of R91. The cells were incubated for 24 h and then stained with PI to evaluate cell death. Percentage killing was quantified by determining the number of PI-stained cells, relative to the total number of cells in the image obtained at 63× magnification using a widefield microscope. Image analyses and cropping were performed using FIJI.

### *S. aureus* infection of macrophages

Efficacy of R91 on intracellular reservoirs of *S. aureus* was determined in a manner as previously described (56). Briefly, R91, at concentrations determined to be noncytotoxic, was added to *in vitro* infections. USA300 LAC was used to infect RAW 264.7 macrophages at an MOI of 10 and incubated for 1.5 h before being treated with 100 μg/ml gentamicin for 1 h to eliminate extracellular bacteria. R91 was subsequently added to the media outside of the infected macrophages, to determine if the compounds were able to restrict the growth of intracellular *S. aureus*. The cells with and without the compounds were then lysed using 0.1% (v/v) Triton X-100 in sterile PBS at 12 h post infection and compared to a sample lysed at the 1.5-h time point immediately after gentamicin treatment to determine the level of bacterial replication by plating on TSA for CFU counts. The fold change in growth was determined by dividing the number of bacteria measured at 12 h post-infection by the number of bacteria that were measured at 1.5 post-infection.

### Mouse SQ abscess model

The mouse study (Animal Use Protocol #2021-090) was approved by the University of Western Ontario Animal Care Committee. Female Balb/c mice were used for this study and were housed in a dedicated mouse facility. The procedure used closely followed that which we have previously published (84). Briefly, eight-to-ten-week-old female Balb/c mice were shaved and naired 24 h prior to infection. Overnight cultures (grown in TSB) of USA300 LAC were subcultured to A_600_ of 0.05 in TSB and grown to A_600_ of 2.0 to 2.2. Bacterial cells were pelleted and washed in PBS twice. Bacterial cells were then normalized to an A_600_ of ∼1.9 (∼4.5–5 × 10^8^ CFUs). Epilated flanks of mice were injected subcutaneously with 50 μl of the bacterial suspension or ∼ 2.25 to 2.5 × 10^7^ CFU in each flank. For control mice, the bacteria were left untreated, whereas for mice receiving R91, the bacteria suspension was treated with R91 at a final concentration of 10 or 50 μM immediately before injection. Mice that received R91 treatment were injected again with 50 μl of PBS containing R91 at 10 or 50 μM 1-day post-infection. The control group received only PBS 1-day post-infection. Infected mice were monitored daily for 3 days and sacrificed at 72 h post infection. Lesions were excised in PBS with 0.1% (v/v) Triton X-100, homogenized, and serially diluted before being plated onto TSA to enumerate CFUs. For each lesion, the bacterial burden was normalized to the mass of the excised lesion. Lesion sizes were analyzed using the ROI measurement tool within the FIJI software package.

### Statistical analyses

All statistical analyses were performed using the Prism9 software package (GraphPad). The number of biological replicates and meaning of error bars can be found in the figure legends. Computational analyses with respect to the RNAseq are described above.

## Data availability

RNAseq data can be found in the GEO repository under accession number GSE266471. The authors declare that the data supporting the findings of this study are available within the paper and its supporting Supplementary information files.

## Supporting information

This article contains supporting information (38, 82).

A document containing Figures S1–S20 and Tables S1 and S2.

Table S3. Excel file containing gene expression levels from RNAseq analysis.

Table S4: Excel file containing ICP-MS output report of metal analysis.

## Conflicts of interest

The authors declare that they have no conflicts of interests with the contents of this article.
