# Supplementary material for: Mechanistic insights and in vivo efficacy of thiosemicarbazones against methicillin-resistant Staphylococcus aureus
Source: J Biol Chem. 2024 Aug 17;300(10):107689. doi: 10.1016/j.jbc.2024.107689 (PMC11492055; doi:10.1016/j.jbc.2024.107689)
Supplement: Supporting information [file mmc1.docx]

**Supplemental Information**

**Figure S1: Chemical structures of R91 and TSC analogs used in this study.** Analogs were selected based on differences in the position and type of substituents on the aromatic rings.

**
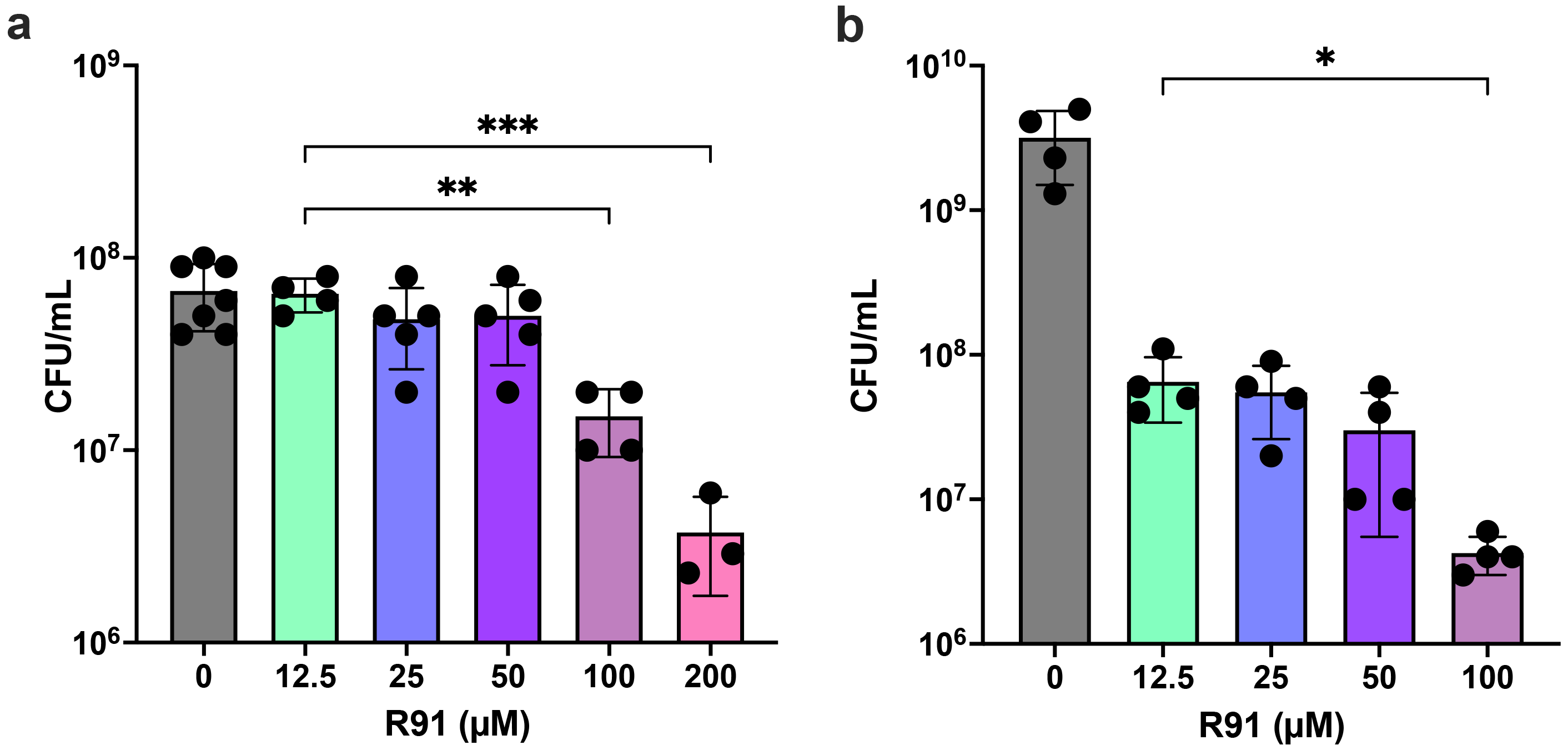
**

**Figure S2: Assessment of bactericidal activity of R91 on MRSA USA300 LAC.** Stationary phase *S. aureus* USA300 was normalized to an OD_600_ of 1 in (a) PBS or (b) MHB with increasing concentrations of R91, starting at the MIC. Bacteria were incubated for 24h before being plated and CFU/mL quantified. Data are shown as the mean ± SD from at least three biological replicates. **p ≤ 0.01, ***p ≤ 0.001 using a one-way ANOVA with Dunnett’s multiple comparisons.


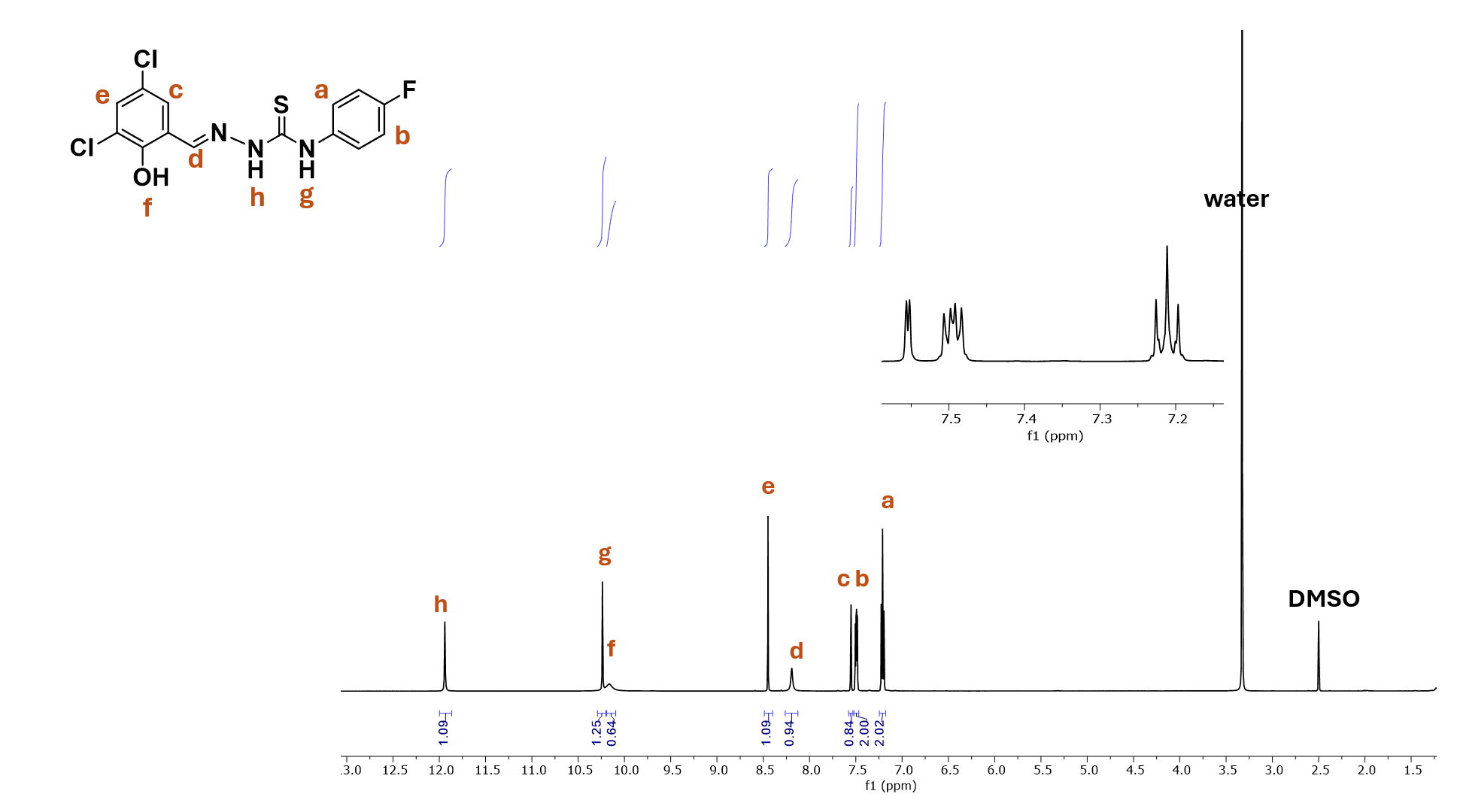


**Figure S3. ^1^H NMR spectrum of R89. Peak integrals, corresponding to the number of associated ^1^H atoms on the molecule, are indicated in blue underneath each peak. The inset corresponds to a zoom-in of the spectrum from 7.1 to 7.6 ppm (600 MHz, DMSO-*d6*).**


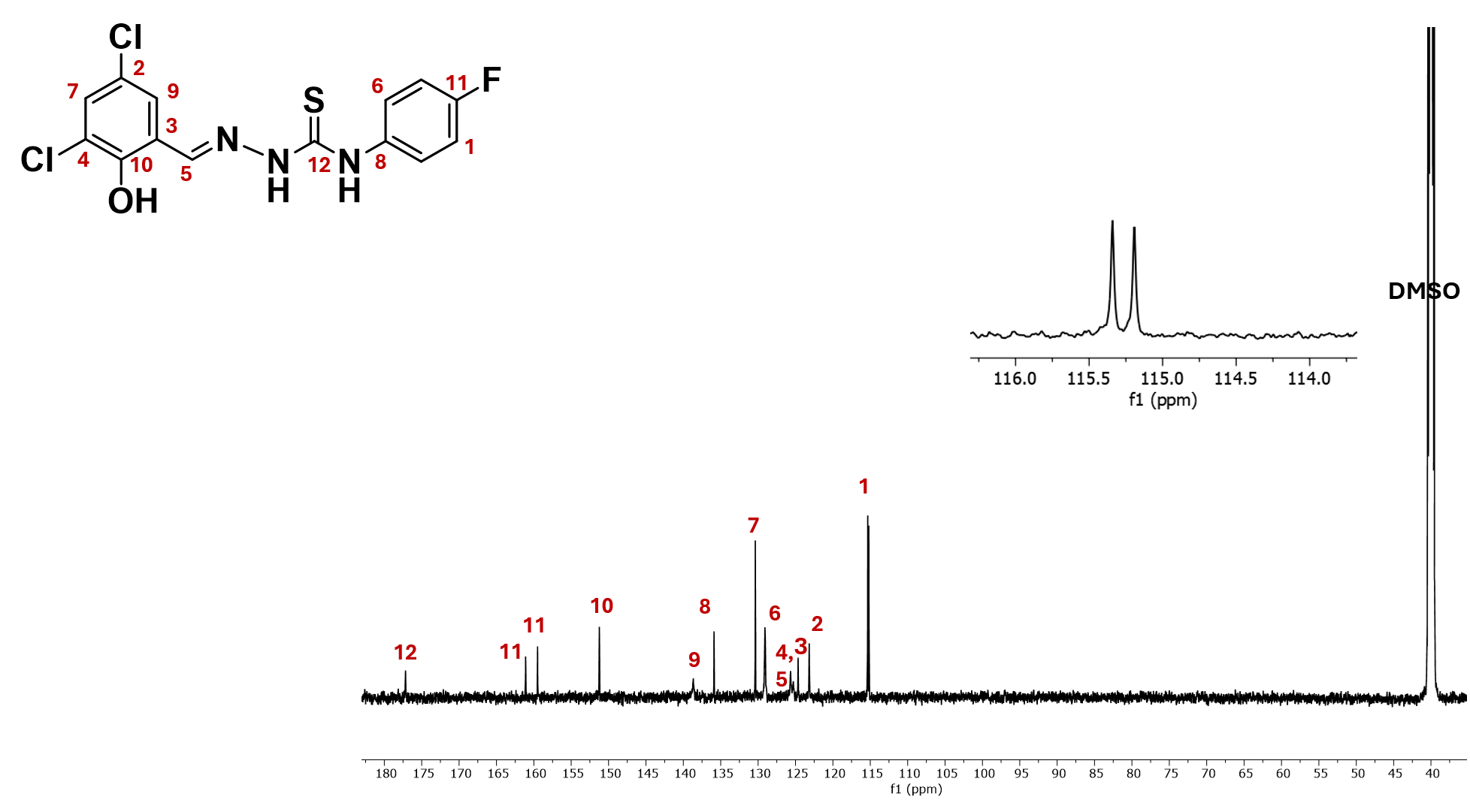


**Figure S4. ^13^C NMR spectrum of R89. The inset corresponds to a zoom-in of the spectrum from 114.0 to 116.0 ppm (600 MHz, DMSO-*d6*).**


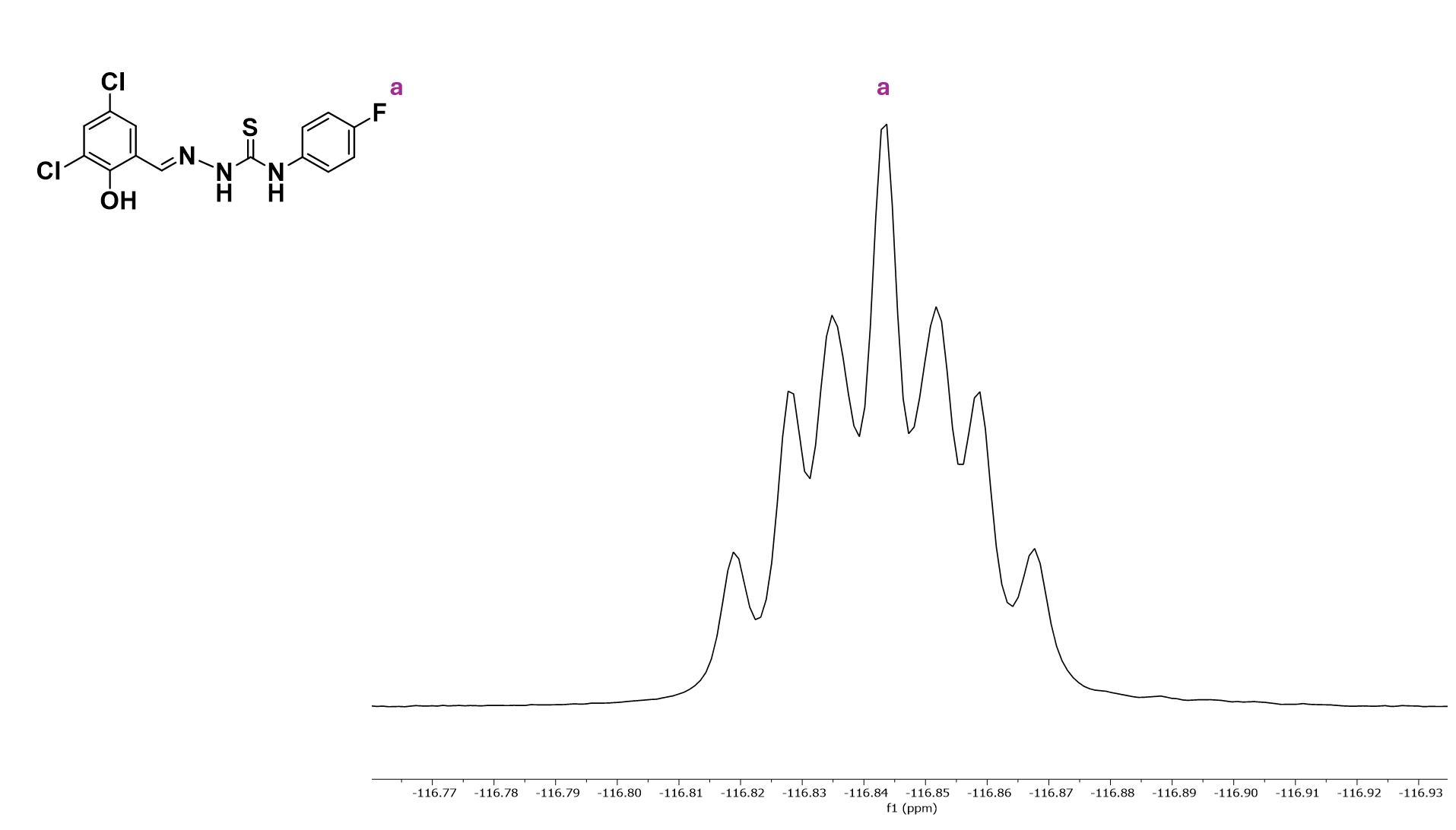


**Figure S5. ^19^F NMR spectrum of R89 (600 MHz, DMSO-*d6*).**


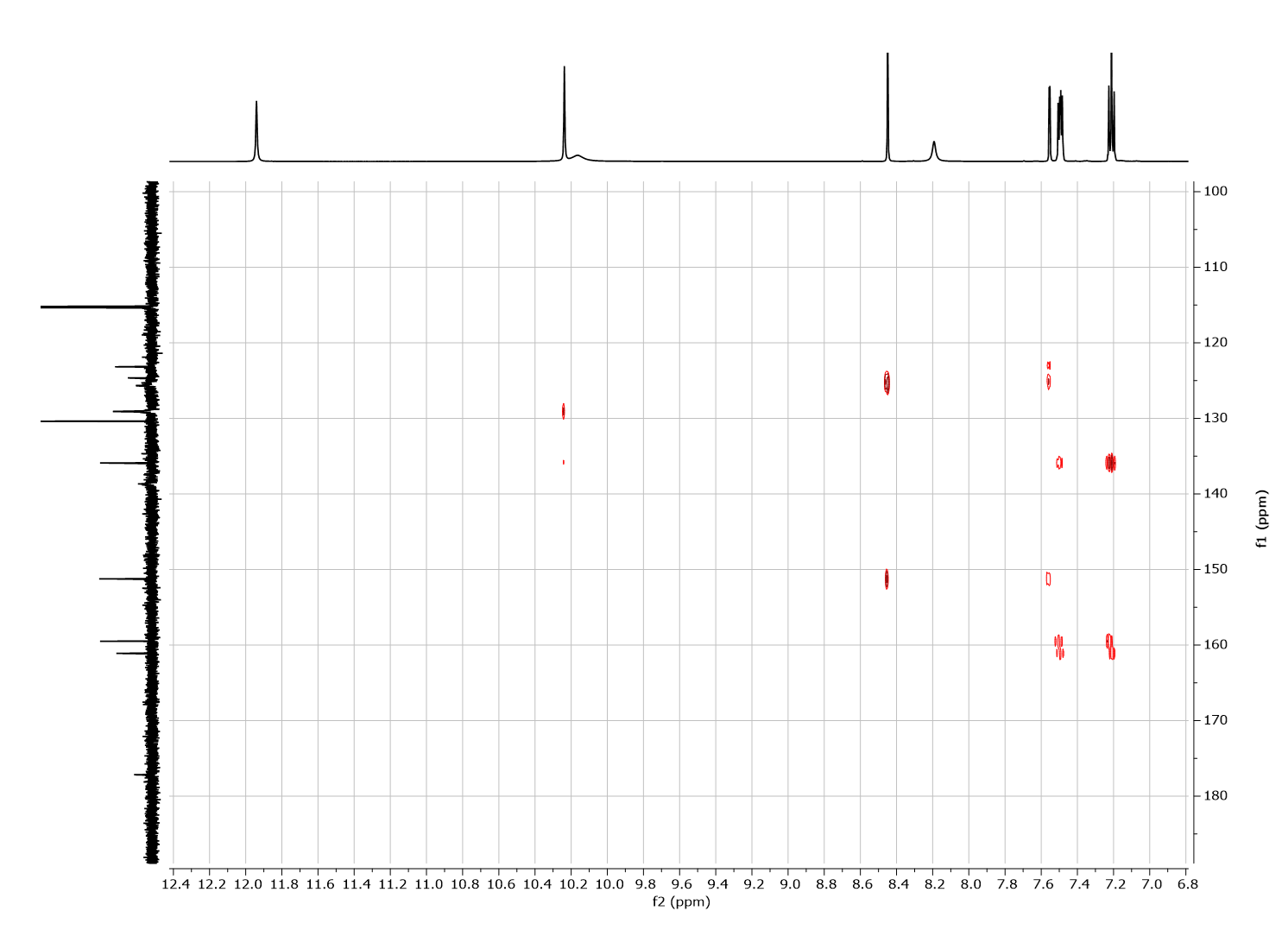


**Figure S6. HMBC NMR spectrum of R89 (600 MHz, DMSO-*d6*).**


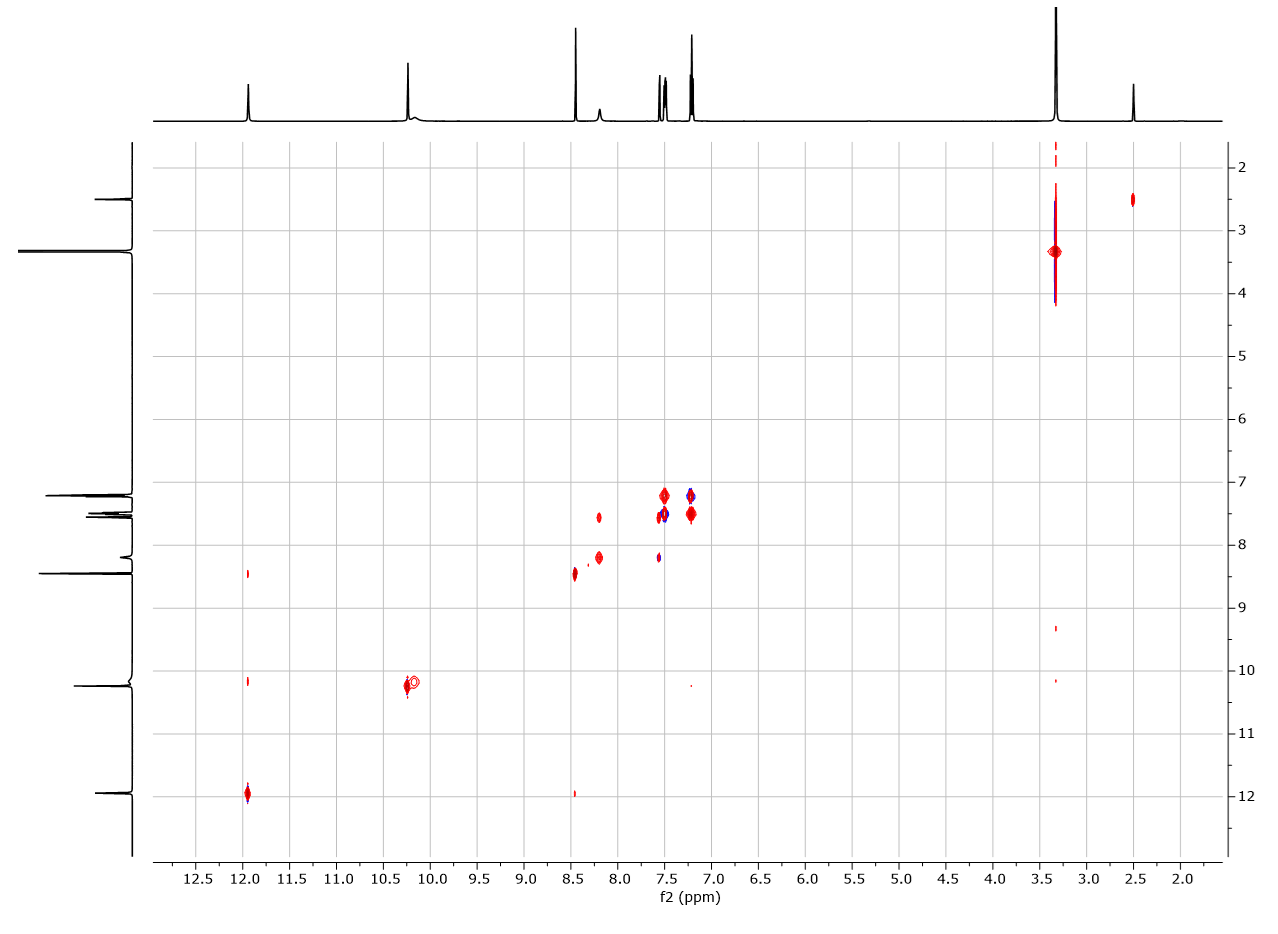


**Figure S7. TOCSY NMR spectrum of R89 (600 MHz, DMSO-*d6*).**


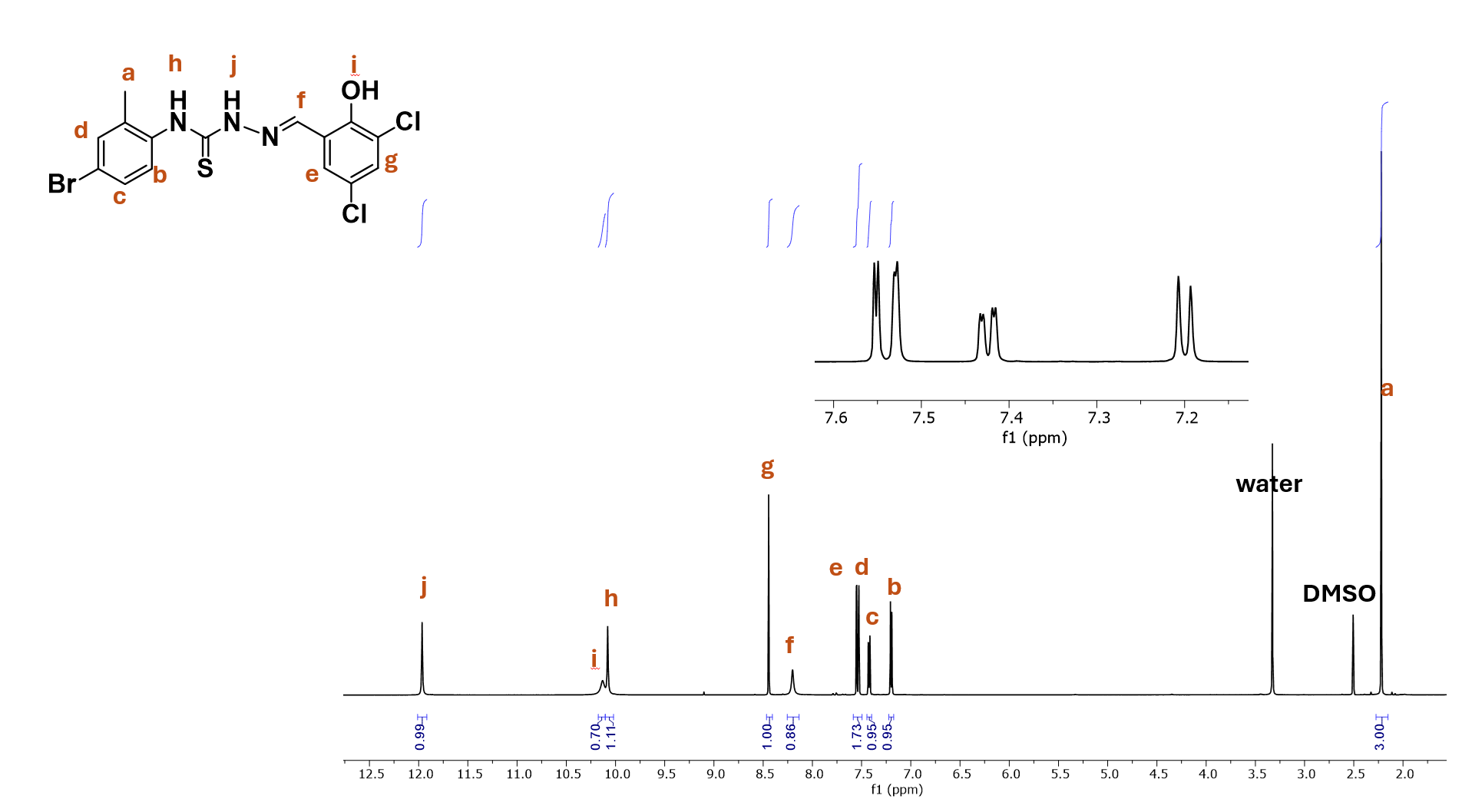


**Figure S8. ^1^H NMR spectrum of R91. Peak integrals, corresponding to the number of associated ^1^H atoms on the molecule, are indicated in blue underneath each corresponding peak. The inset corresponds to a zoom-in of the spectrum from 7.1 to 7.6 ppm (600 MHz, DMSO-*d6*).**


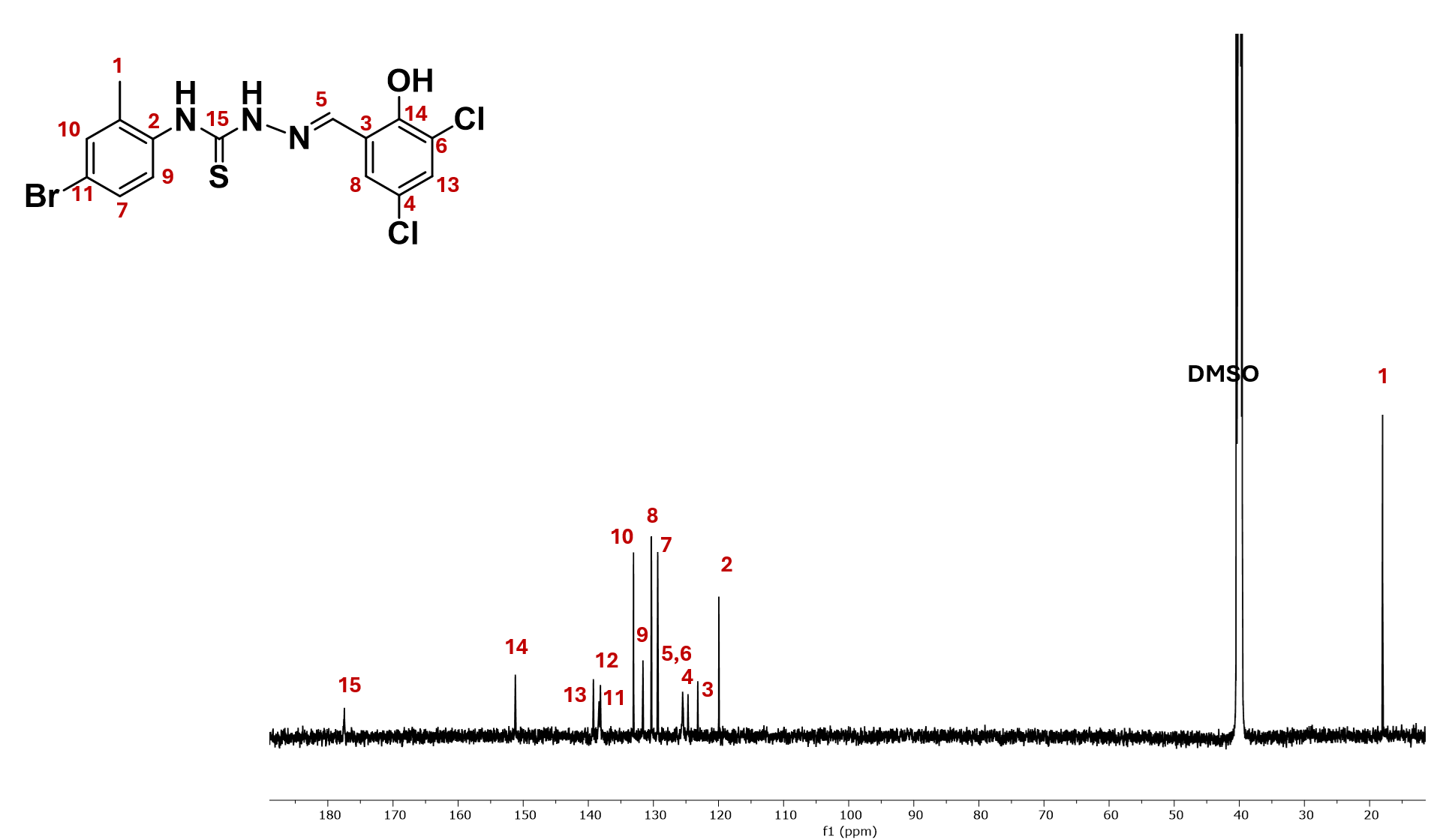


**Figure S9. ^13^C NMR spectrum of R91 (600 MHz, DMSO-*d6*).**


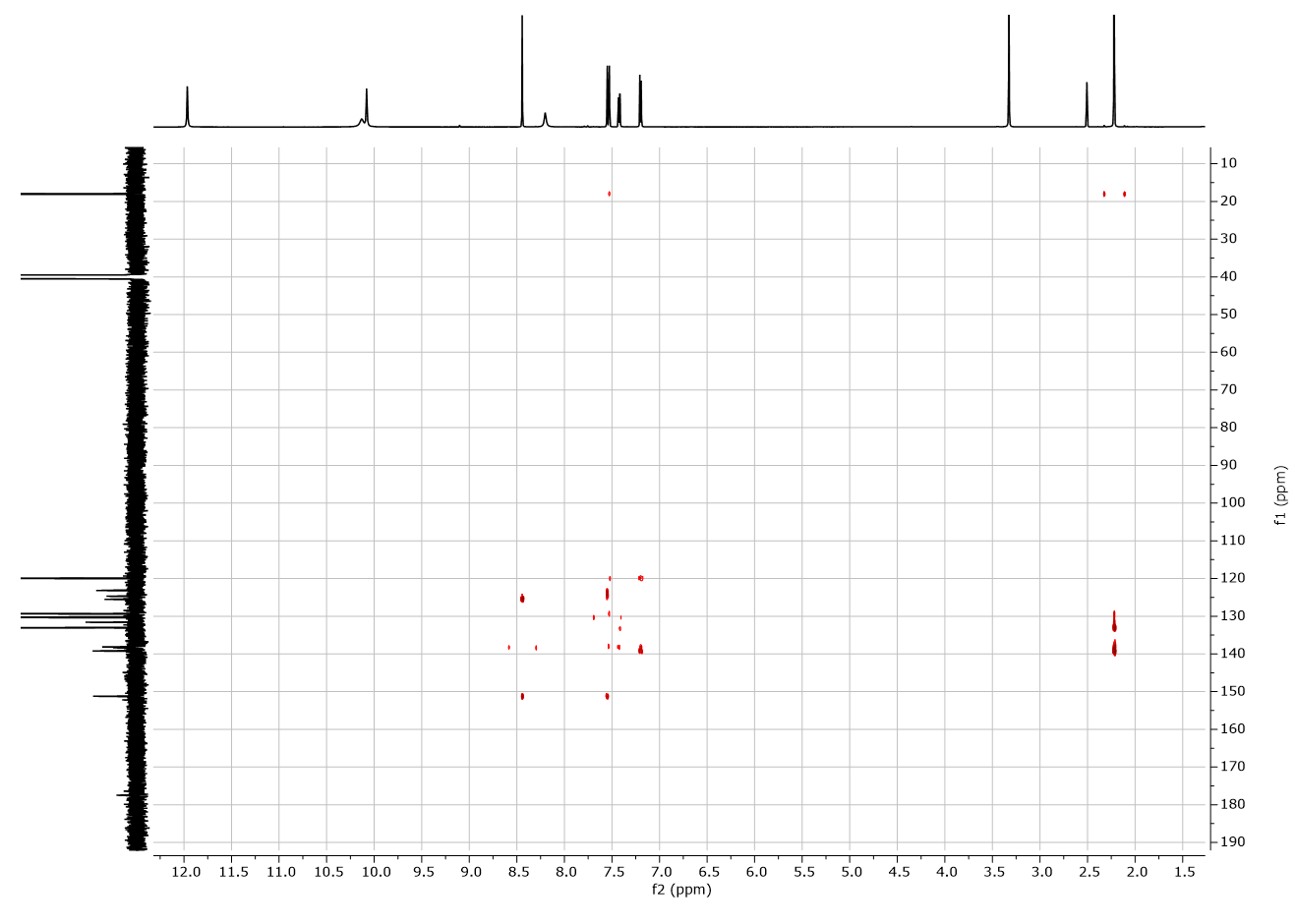


**Figure S10. HMBC NMR spectrum of R91 (600 MHz, DMSO-*d6*).**


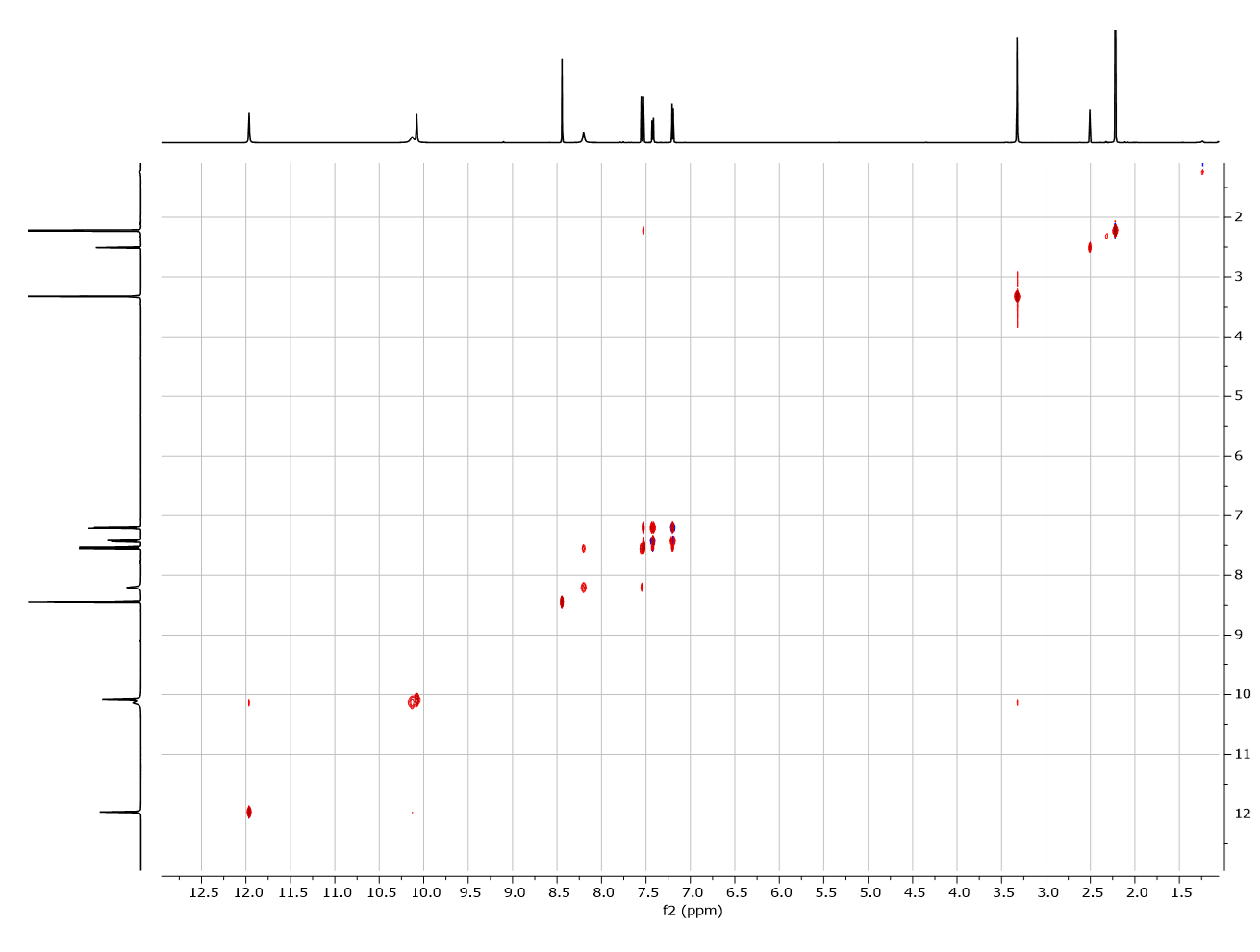


**Figure S11. TOCSY NMR spectrum of R91 (600 MHz, DMSO-*d6*).**


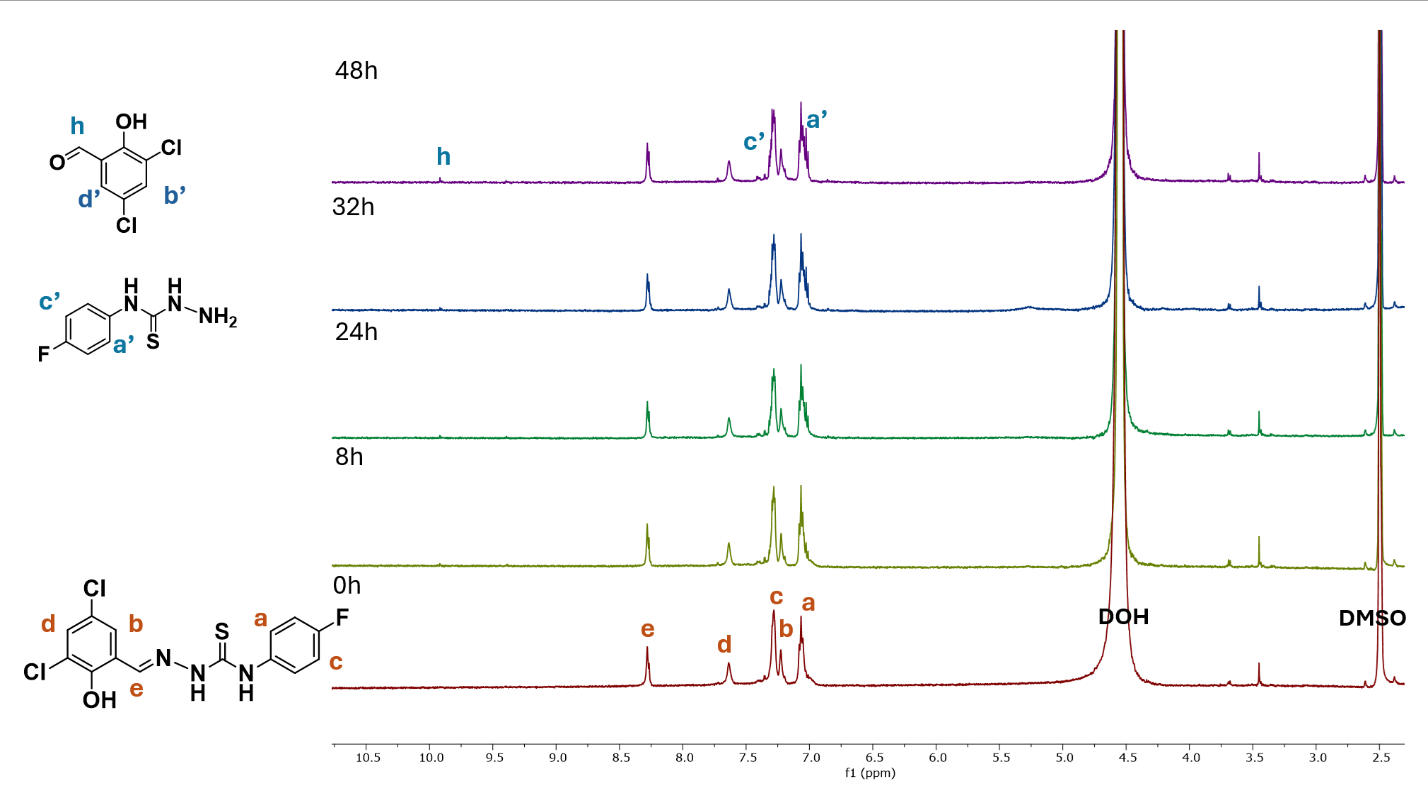
**Figure S12: Stability testing of R89.** ^1^H NMR spectra of R89 in 5:1 phosphate buffered D_2_O (0.01 M pH 7.4): DMSO-*d_6_* (5:1) incubated at 37 ^o^C for 48 hours.


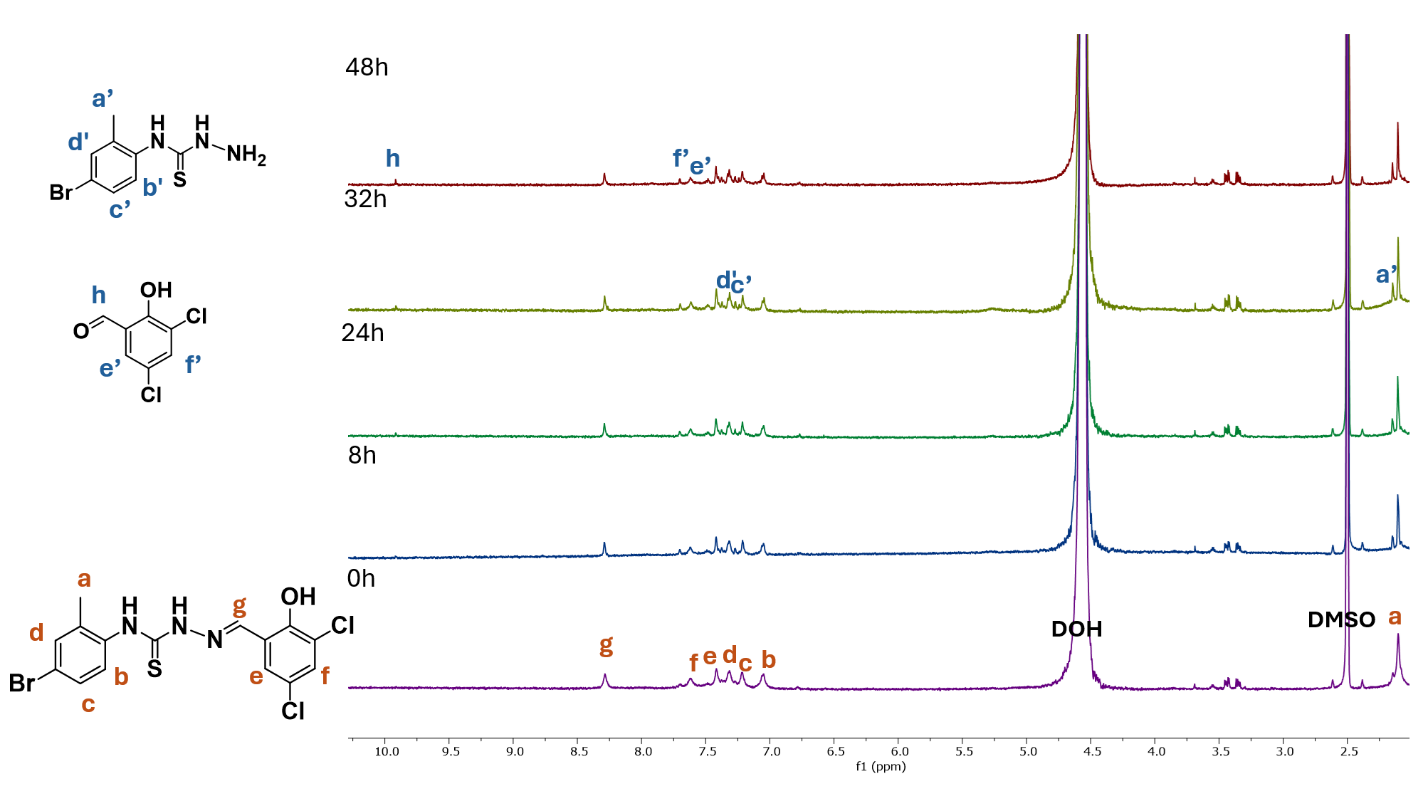


**Figure S13: Stability testing of R91.** ^1^H NMR spectra of R91 in 0.01 M pH 7.4 phosphate buffer (D_2_O): DMSO-d6 (5:1) incubated at 37 ºC over 48 hours.


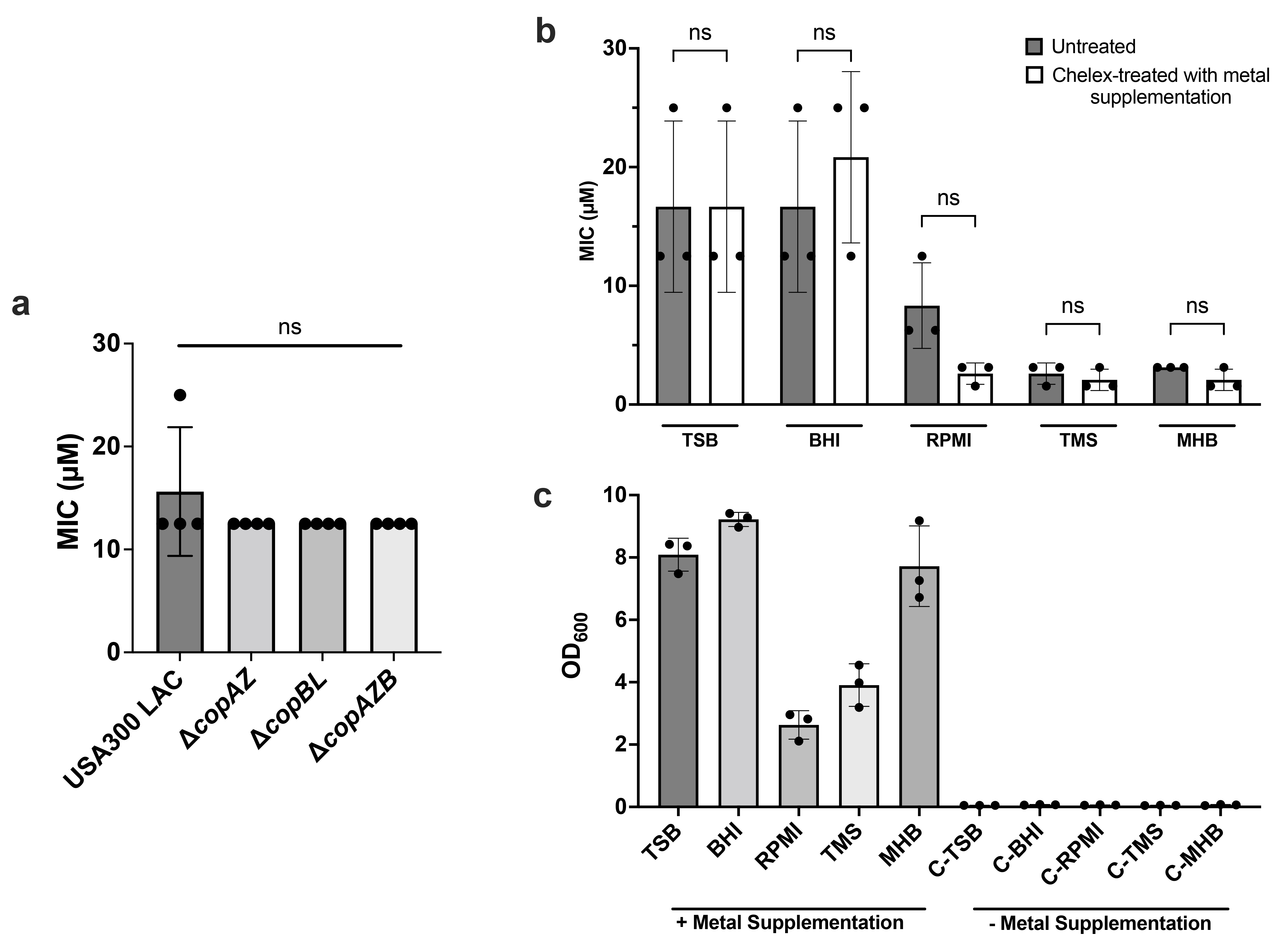


**Figure S14: Copper export deficiency and metal chelation do not alter the MIC of R91.** (a) MIC of R91 against *S. aureus* USA300 LAC and various copper-export deficient mutants in TSB. Data are shown as the mean ± SD from at least three biological replicates. Data were not significant (ns) using a one-way ANOVA with Dunnett’s multiple comparisons. (b) MIC of USA300 LAC in Chelex-100 treated and untreated media. Chelex-100 treated media was supplemented with 50μM FeSO_4_, 50μM MnCl_2_ and 50μM CaCl_2_. Data are shown as the mean ± SD from at least three biological replicates. Data were not significant (ns) using a one-way ANOVA with Šidák’s multiple comparison test with a single pooled variance. (c) MIC of USA300 LAC in Chelex-100 treated media with and without Fe, Mn, and Ca supplementation. Without metal supplementation there is no bacterial growth, indicating Chelex-100 treatment successfully removed essential trace metals.


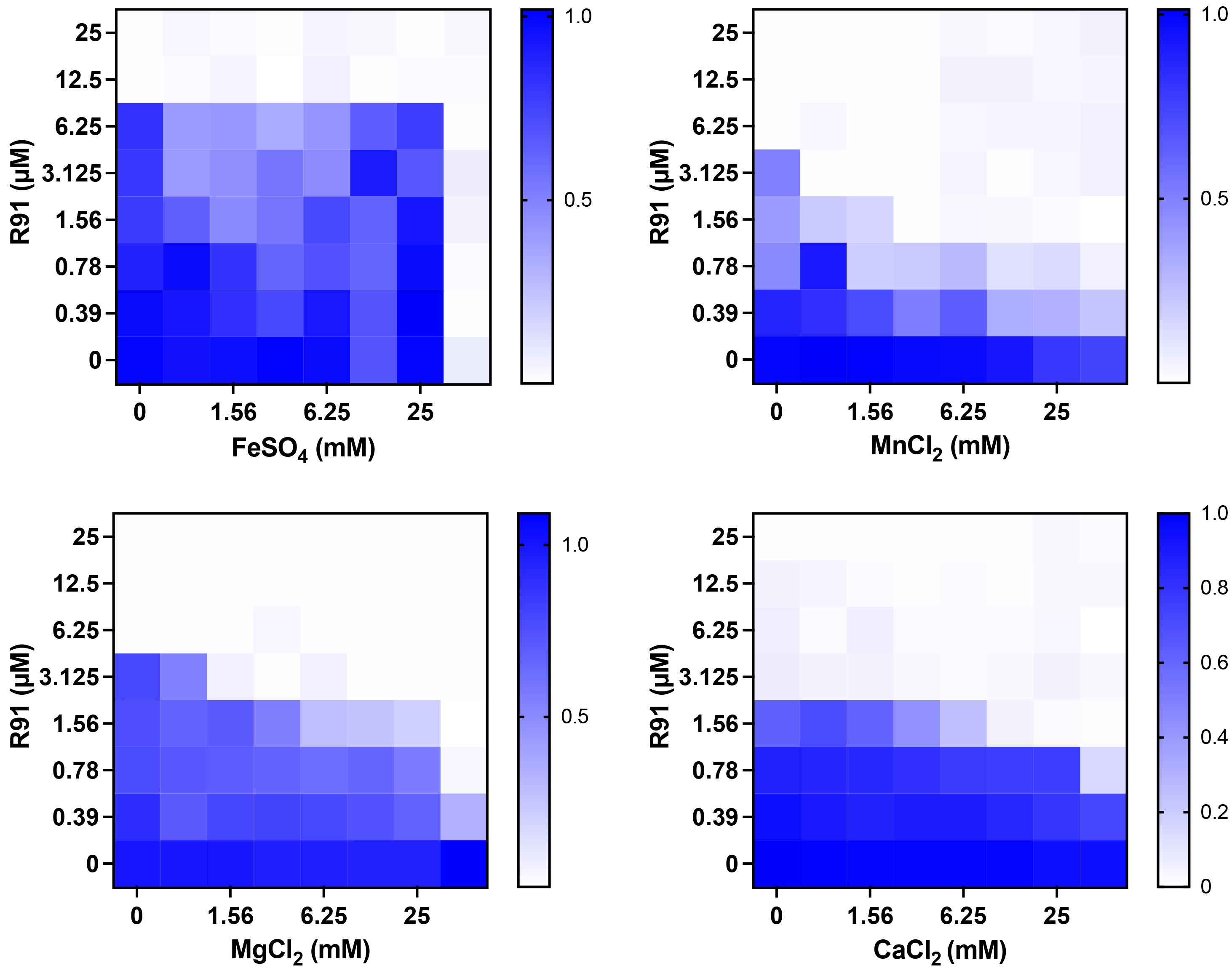


**Figure S15: Anti-MRSA activity of R91 is unaffected by Fe^2+^, Mn^2+^, Mg^2+^ and Ca^2+^.** Representative checkerboard assays of R91 with FeSO_4_, MgCl_2_, MnCl_2_ or CaCl_2_ against *S. aureus* USA300 LAC. Growth was quantified by measuring absorbance at OD_600_, and results are represented using heat maps, where a darker blue color corresponds to a greater bacterial density. Checkerboards were completed at metal concentrations significantly higher than physiologically relevant, or what was used for synergistic/antagonistic metals, however, the MIC of Mn^2+^, Mg^2+^ and Ca^2+^ was not reached, and thus FICI could not be quantified. Indifference was instead determined by the overall pattern of the checkerboards, which corresponds to the typical or expected trend and shape of indifferent or additive interactions. The checkerboards were repeated three times independently and all replicates yielded similar results.


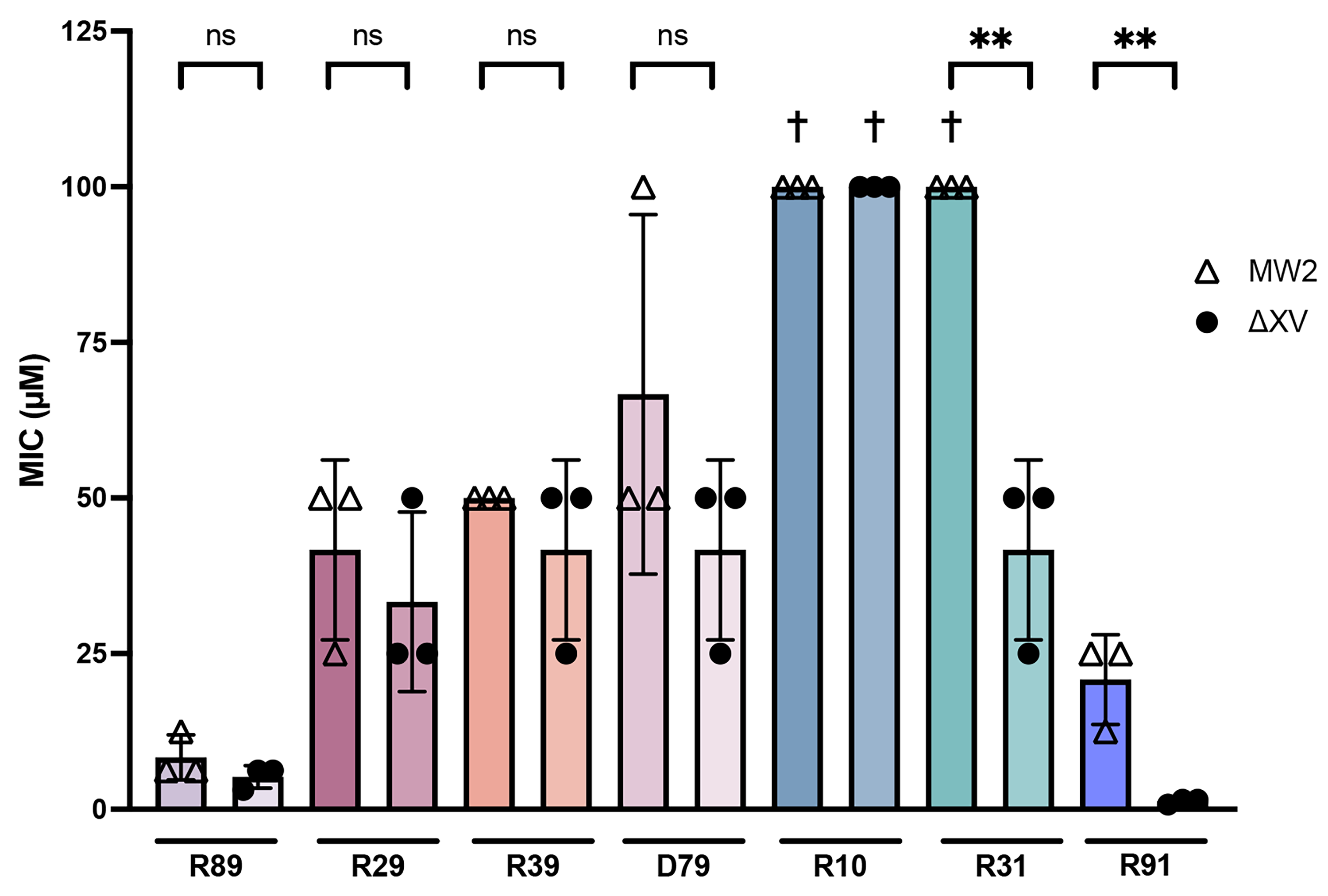


**Figure S16: *S. aureus* MW2 ΔXV is not hypersensitive to most R91 analogs.** MIC of R91 and analogs against the *S. aureus* MW2 and its isogenic ΔXV mutant. The highest concentration of drug tested was 100μM. † indicates the MIC is above the highest concentration tested. Data are shown as the mean ± SD from three biological replicates. **p ≤ 0.01 using an unpaired t-test comparing the MIC of each analog on MW2 to the MIC of that analog on ΔXV.


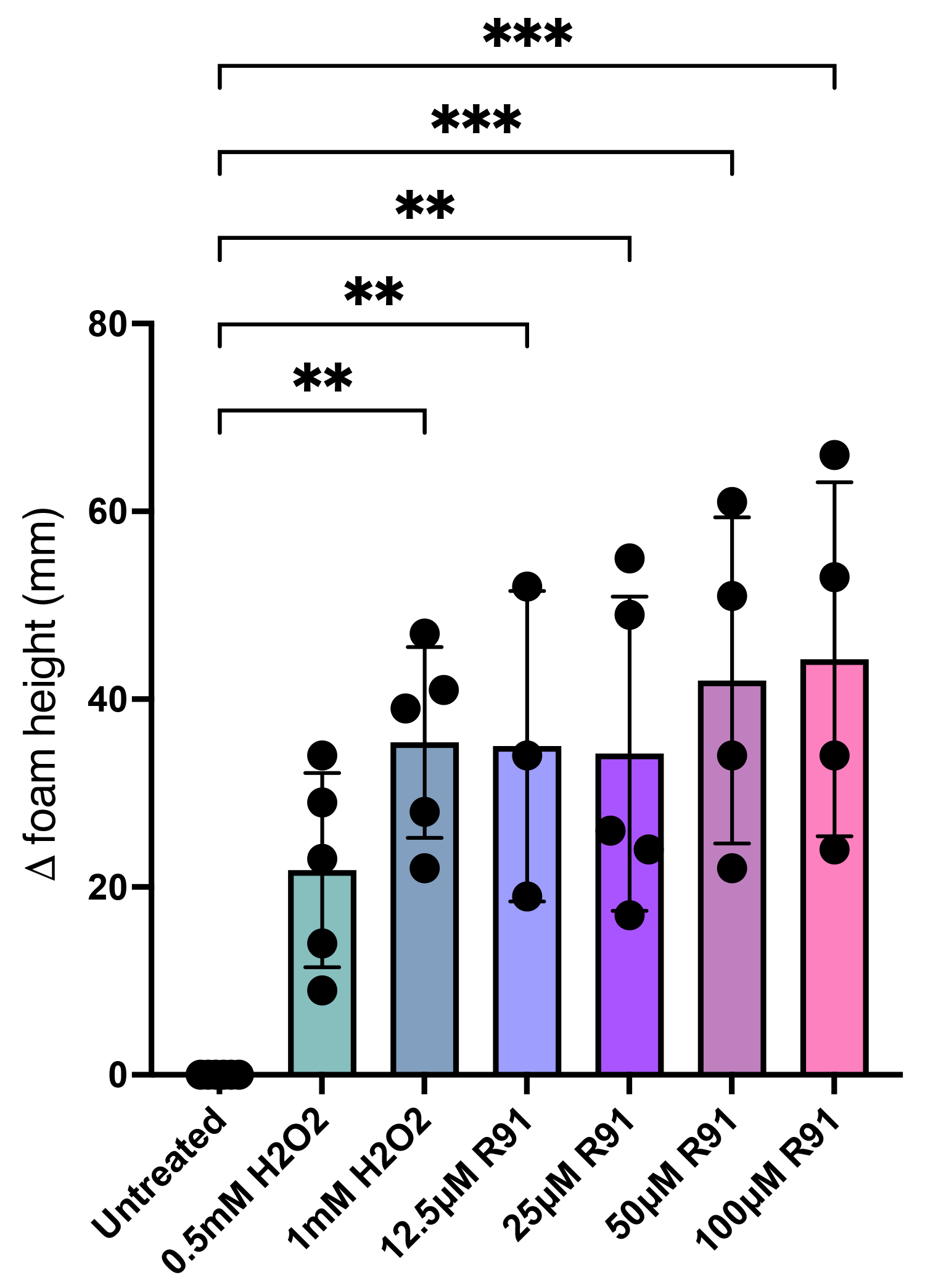


**Figure S17: R91 exposure increases *S. aureus* catalase activity.** Relative catalase activity was measured using a change in the height of foam produced by the decomposition of hydrogen peroxide by catalase. Foam heights were measured in Pyrex tubes using a ruler and normalized to the height of the untreated control. Data are shown as the mean ± SD of at least three independent experiments. **p ≤ 0.01, ***p ≤ 0.001 using a one-way ANOVA with Dunnett’s multiple comparison.


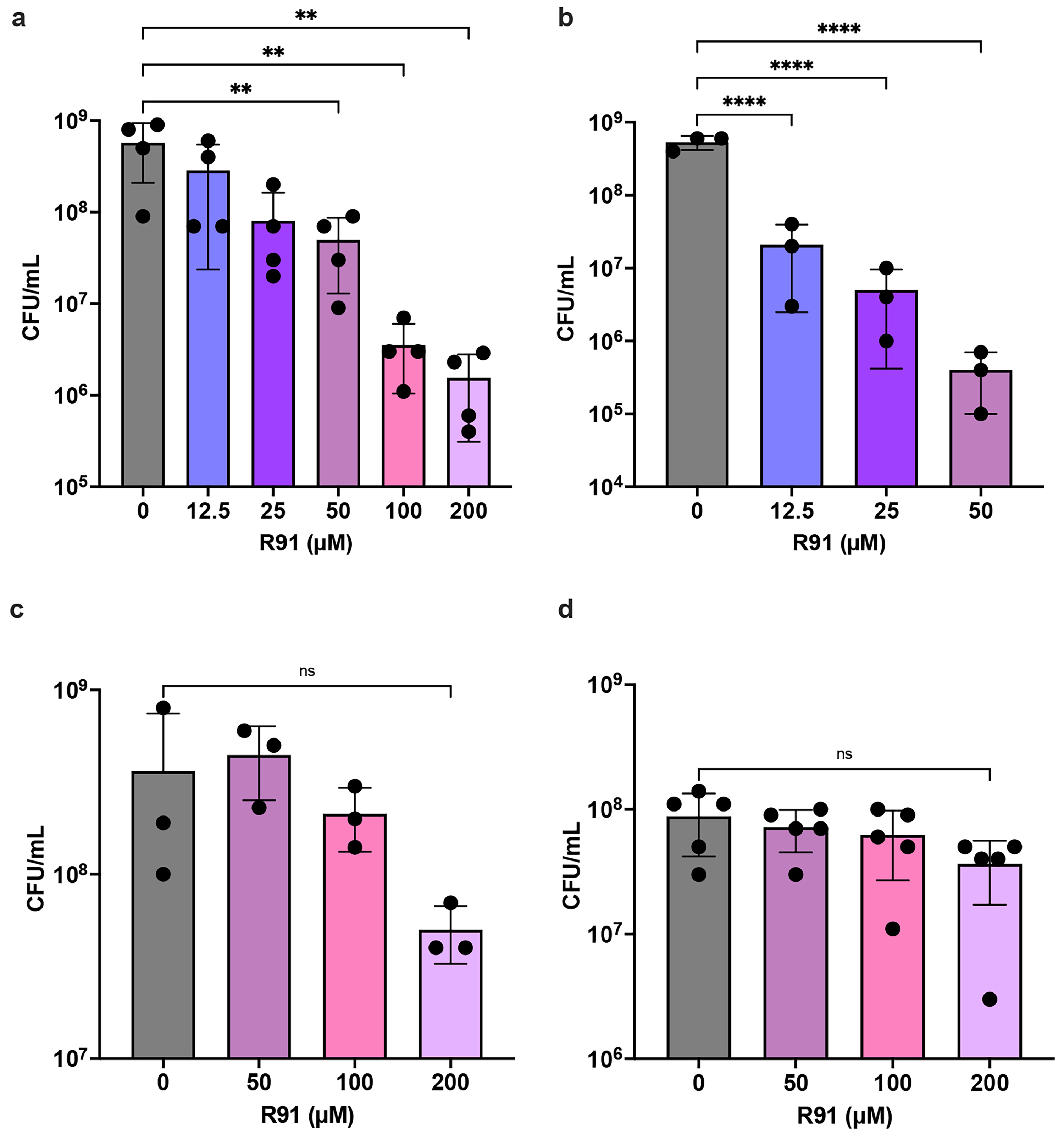


**Figure S18:** **Hydrogen peroxide defense is important for R91 resistance.** Killing by R91 of strains (a) *S. aureus* USA300 LAC, (b) USA300 *katA*::tn, (c) USA300 *perR*::tn and (d) USA300 + p*katA*. Stationary phase *S. aureus* was normalized to an OD_600_ of 1 in PBS with various concentrations of R91. The bacteria were incubated for 24h before being plated and CFU/mL quantified. Data are shown as the mean ± SD from three biological replicates. **p ≤ 0.01, ****p ≤ 0.0001 using a one-way ANOVA with Dunnett’s multiple comparisons.

**Figure S19: Resistance to R91 could not be acquired after exposure to drug.** Bacteria were serially passaged over a 14-day period in TSB containing ½ MIC of either R91 or ciprofloxacin. The MIC of R91 and ciprofloxacin (positive control) were determined for each successful passage. Day 0 corresponds to the parental strain (*S. aureus* USA300 LAC) before challenge with each antibiotic. Figure shows the MIC fold change of the passaged cultures compared to the parental strain. The starting MIC of R91 and ciprofloxacin were 12.5μM and 50μg/mL, respectively.


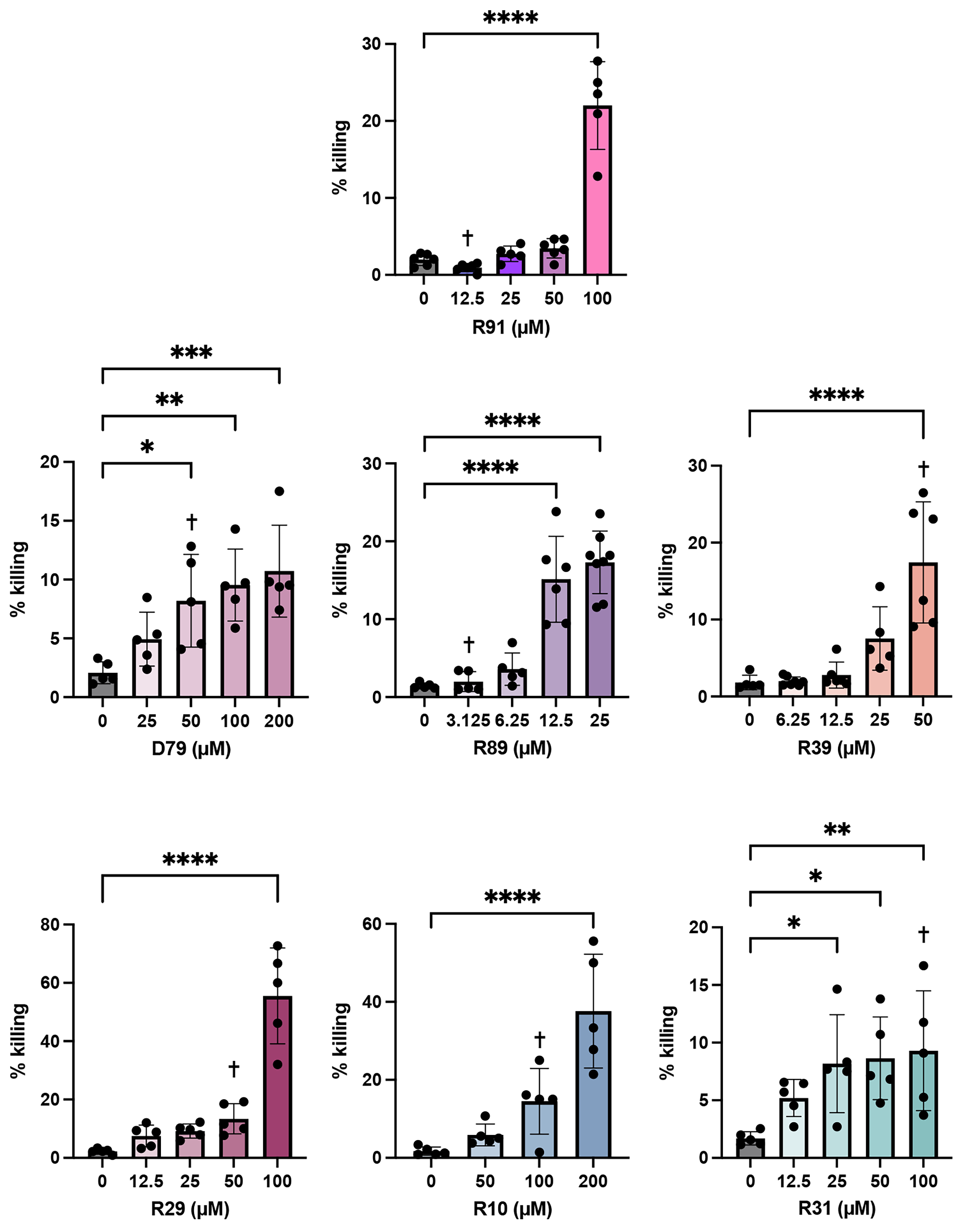


**Figure S20: R91 is less cytotoxic at effective concentrations compared to most analogs.** Analogs were incubated with RAW264.7 macrophages in RPMI supplemented with 5% fetal bovine serum (FBS). After 24h the cells were stained with propidium iodide (PI) and imaged using a widefield microscope. The % macrophage killing was calculated using the number of PI-stained cells compared to the total number of cells. The MIC (μM) of each TSC against USA300 LAC is indicated by †. Data shown are means ± SD of at least five independent experiments. Data and the graph corresponding to R91 are duplicated from Figure 7b for comparative purposes. *p ≤ 0.05, **p ≤ 0.01, ***p ≤ 0.001, ****p ≤ 0.0001 using a one-way ANOVA with Dunnett’s multiple comparison.

**Table S1: Bacterial strains used in this study**

| **Strain or Plasmid** | **Description** | **Source or reference** |
| --- | --- | --- |
| ***S. aureus*** | | |
| USA300 | USA300 LAC, cured of resistance plasmids | Lab stock |
| RN4220 | r_K_^−^ m_K_^+^; capable of accepting foreign DNA | Lab stock |
| USA100 | WT *S. aureus* USA100 strain | Lab stock |
| BK21203 | WT *S. aureus* USA200 strain, MN8 lineage | Lab stock |
| MW2 | WT *S. aureus* USA400 strain | Lab stock |
| USA600 | WT *S. aureus* USA600 strain | Lab stock |
| MW2 ΔXV | Strain MW2 with complete deletion of 15 non-essential two-component systems | *38* |
| MW2 Δ*vraRS* | Strain MW2 with complete deletion of *vraRS* | *38* |
| MW2 Δ*graRS* | Strain MW2 with complete deletion of *graRS* | *38* |
| MW2 Δ*arlRS* | Strain MW2 with complete deletion of *arlRS* | *38* |
| MW2 Δ*saeRS* | Strain MW2 with complete deletion of *saeRS* | *38* |
| MW2 Δ*hptRS* | Strain MW2 with complete deletion of *hptRS* | *38* |
| MW2 Δ*airRS* | Strain MW2 with complete deletion of *airRS* | *38* |
| MW2 Δ*phoRP* | Strain MW2 with complete deletion of *phoRP* | *38* |
| MW2 Δ*yhcSR* | Strain MW2 with complete deletion of *yhcSR* | *38* |
| MW2 Δ*agr* | Strain MW2 with complete deletion of *agr* | *38* |
| MW2 Δ*kdpDE* | Strain MW2 with complete deletion of *kdpDE* | *38* |
| MW2 Δ*hssRS* | Strain MW2 with complete deletion of *hssRS* | *38* |
| MW2 Δ*nreBC* | Strain MW2 with complete deletion of *nreBC* | *38* |
| MW2 Δ*braRS* | Strain MW2 with complete deletion of *braRS* | *38* |
| MW2 Δ*lytSR* | Strain MW2 with complete deletion of *lytSR* | *38* |
| MW2 Δ*srrAB* | Strain MW2 with complete deletion of *srrAB* | *38* |
| MW2 Δ*srrAB* p*srrAB* | Strain MW2 with complete deletion of *srrAB*, carrying the wild-type *srrAB* from MW2 | This study |
| MW2 Δ*srrAB* pEmpty | Strain MW2 with complete deletion of *srrAB*, carrying empty pALC2073 | This study |
| USA300 Δ*menD* | Strain MW2 with complete deletion of *menD* | This study |
| USA300 Δ*menD qoxA*::tn | Strain MW2 with complete deletion of *menD* and transposon insertion of *qoxA* | This study |
| USA300 Δ*menD* Δ*cydAB* | Strain MW2 with complete deletion of *menD* and *cydAB* | This study |
| USA300 Δ*menD* Δ*cydAB qoxA*::tn | Strain MW2 with complete deletion of *menD* and *cydAB* and transposon insertion of *qoxA* | This study |
| USA300 Δ*sodAM* | WT *S. aureus* USA300 with complete deletion of *sodAM* | Lab stock |
| USA300 *katA*::tn | WT *S. aureus* USA300 with a transposon mutation of *katA* | NTML library |
| USA300 *perR*::tn | WT *S. aureus* USA300 with a transposon mutation of *perR* | NTML library |
| USA300 p*katA* | WT *S. aureus* USA300 carrying the wild-type *katA* gene from USA300 on plasmid pALC2073 | This study |
| **Other Staphylococcal species** | | |
| *S. epidermidis* Mach 1457 | Competent clinical strain | Lab stock |
| *S. epidermidis* M23864:W2 (HM-144) | Human skin isolate | Lab stock |
| *S. lugdunensis* M23590 (HM-141) | Human skin isolate | ATCC type strain |
| *S. saprophyticus* ATCC 15305 | Urine isolate | ATCC type strain |
| *S. chromogenes* ATCC 43764 | From the original ATCC stock culture , this isolate possesses a white, mucoid colony | ATCC type strain |
| *S. capitis* ATCC 35661 | Human skin isolate | ATCC type strain |
| *S. cohnii* ATCC 29973 | Human skin isolate | ATCC type strain |
| **Other Gram-positive bacteria** | | |
| *M. luteus* ATCC 4698 | Human nasal secretion isolate | ATCC type strain |
| *B. subtilis* 3A1T | Wild-type isolate | Bacillus Genetic Stock Center |
| *S. pyogenes* MGAS8232 | Isolated from a patient with acute rheumatic fever. | J. McCormick |
| *S. agalactiae* A909 | Isolated from a septic human neonate | ATCC type strain |
| *E. faecalis* ATCC 33186 | Strain CN478; historical urine isolate | ATCC type strain |
| **Gram-negative bacteria** |  |  |
| *P. aeruginosa* PAO1 | Wildtype strain | K. Poole |
| *E. coli* DH5α | F^−^ ϕ80d*lacZ*ΔM15 *recA1 endA1 gyrA96 thi-1 hsdR17*(r_K_^−^ m_K_^−^) *supE44 relA1 deoR* Δ(*lacZYA-argF*)*U169 phoA* | Promega |
| **Plasmids** | | |
| pALC2073 | *E. coli - S. aureus* shuttle vector. Amp^R^ in *E. coli*, Cm^R^ in *S. aureus* | *82* |

**Table S2: Oligonucleotides used in this study**

| **Name** | **Sequence** | **Description** |
| --- | --- | --- |
| srrB | TATATAGAGCTCAATTTTATTCTGGTTTTG | Forward primer to amplify *srrAB* operon from *S. aureus* USA400 MW2 |
| srrA | TATATAGGTACCTGTGTGGGAGGTATGACC | Reverse primer to amplify *srrAB* operon from *S. aureus* USA400 MW2 |
| katA_F | TATATAGGTACCAATTATTATAAATTGTGGAGGG | Forward primer to amplify *katA* from *S. aureus* USA300 LAC |
| katA_R | TATATAGAGCTCACTAGATATCAAATTTATTTTTCAAAG | Reverse primer to amplify *katA* from *S. aureus* USA300 LAC |
| *menD* pKOR1 UpF | GGGGACAAGTTTGTACAAAAAAGCAGGCT AATTATCACAGTCACTAGACCCGAG | Forward primer for upstream region of *menD* from *S. aureus* USA300 LAC |
| *menD* pKOR1 UpR | GGACCTCCGCGGTCTTTACTATCATCCGCAATAGGCA | Reverse primer for upstream region of *menD* from *S. aureus* USA300 LAC |
| *menD* pKOR1 DnF | GGACCTCCGCGGGCCACATTGTTATCTGAAACTTCGA | Forward primer for downstream region of *menD* from *S. aureus* USA300 LAC |
| *menD* pKOR1 DnF | GGGGACCACTTTGTACAAGAAAGCTGGGT ATTCAATTTCTCACCAGCTCGTTTT | Reverse primer for downstream region of *menD* from *S. aureus* USA300 LAC |
| *cydAB* pKOR1 UpF | GGGGACAAGTTTGTACAAAAAAGCAGGCT GTATGTCAGAGCGTGTATCA | Forward primer for upstream region of *cydAB* from *S. aureus* USA300 LAC |
| *cydAB* pKOR1 UpR | GGACCTCCGCGGAAACATCACCTTTTTCTTTTTTTGA | Reverse primer for upstream region of *cydAB* from *S. aureus* USA300 LAC |
| *cydAB* pKOR1 DnF | GGACCTCCGCGGTAAGTTGAGACGATACCCCA | Forward primer for downstream region of *cydAB* from *S. aureus* USA300 LAC |
| *cydAB* pKOR1 DnF | GGGGACCACTTTGTACAAGAAAGCTGGGT TTTCAATTCTAGTCAGGGGC | Reverse primer for downstream region of *cydAB* from *S. aureus* USA300 LAC |
